# Plutonic xenoliths from Martinique, Lesser Antilles: evidence for open system processes and reactive melt flow in island arc crust

**DOI:** 10.1007/s00410-016-1299-8

**Published:** 2016-09-27

**Authors:** George F. Cooper, Jon P. Davidson, Jon D. Blundy

**Affiliations:** 1 Science Labs, Department of Earth Sciences, Durham University, Durham, DH1 3LE UK; 2 School of Earth Sciences, University of Bristol, Wills Memorial Building, Bristol, BS8 1RJ UK

**Keywords:** Lesser Antilles, Martinique, Plutonic, Xenoliths, Amphibole, Reactive melt

## Abstract

The Lesser Antilles Volcanic Arc is remarkable for the abundance and variety of erupted plutonic xenoliths. These samples provide a window into the deeper crust and record a more protracted crystallisation history than is observed from lavas alone. We present a detailed petrological and in situ geochemical study of xenoliths from Martinique in order to establish their petrogenesis, pre-eruptive storage conditions and their contribution to construction of the sub-volcanic arc crust. The lavas from Martinique are controlled by crystal–liquid differentiation. Amphibole is rarely present in the erupted lavas, but it is a very common component in plutonic xenoliths, allowing us to directly test the involvement of amphibole in the petrogenesis of arc magmas. The plutonic xenoliths provide both textural and geochemical evidence of open system processes and crystal ‘cargos’. All xenoliths are plagioclase-bearing, with variable proportions of olivine, spinel, clinopyroxene, orthopyroxene and amphibole, commonly with interstitial melt. In Martinique, the sequence of crystallisation varies in sample type and differs from other islands of the Lesser Antilles arc. The compositional offset between plagioclase (~An_90_) and olivine (~Fo_75_), suggests crystallisation under high water contents and low pressures from an already fractionated liquid. Texturally, amphibole is either equant (crystallising early in the sequence) or interstitial (crystallising late). Interstitial amphibole is enriched in Ba and LREE compared with early crystallised amphibole and does not follow typical fractionation trends. Modelling of melt compositions indicates that a water-rich, plagioclase-undersaturated reactive melt or fluid percolated through a crystal mush, accompanied by the breakdown of clinopyroxene, and the crystallisation of amphibole. Geothermobarometry estimates and comparisons with experimental studies imply the majority of xenoliths formed in the mid-crust. Martinique cumulate xenoliths are inferred to represent crystal mushes within an open system, through which melt can both percolate and be generated.

**Electronic supplementary material:**

The online version of this article (doi:10.1007/s00410-016-1299-8) contains supplementary material, which is available to authorized users.

## Introduction

**Fig. 1:**
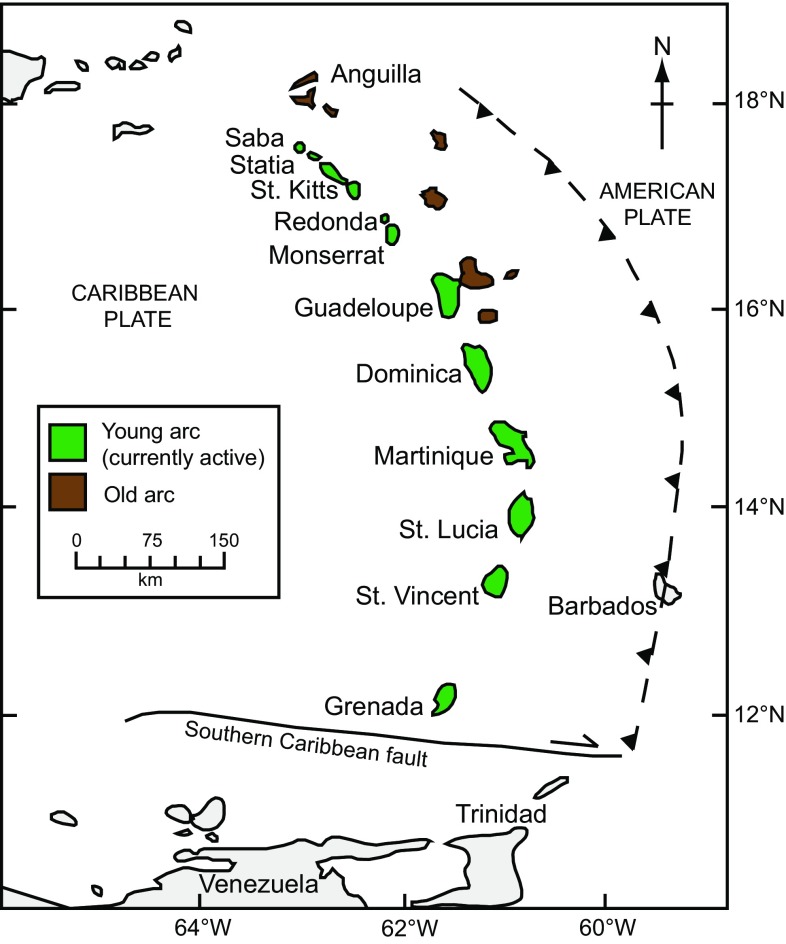
Map of the Lesser Antilles Island arc. The *dashed line* depicts the approximate position of the subduction zone (after Wadge and Shepherd 1984). Islands making up the old arc (*brown*) and the currently active arc (*green*) are denoted

Arc magmas are commonly highly differentiated and rarely represent primary mantle-derived melts. The vast majority of studies on arc magmatism are restricted to samples of the extrusive products, which represent the end products of magmatic processes that may occur over considerable time and depth ranges within the arc crust. On the other hand, plutonic xenoliths, representing erupted plutonic samples, have a greater preservation potential than phenocrysts in lavas and are therefore more likely to provide a window into the true fractionation history of magmas (Arculus and Wills 1980; Macdonald et al. 2000). The Lesser Antilles Arc is exceptional globally in respect to the abundance and variety of erupted plutonic xenoliths, which are the focus of several studies (Lewis 1973; Arculus and Wills 1980; Tollan et al. 2012; Stamper et al. 2014). Therefore, the Lesser Antilles is an ideal location to study the relationship between extrusive and intrusive components of an arc magmatic system. Our focus is the island of Martinique in the centre of the Lesser Antilles arc (Fig. 1). We present a detailed petrological, mineralogical and in situ geochemical study of a diverse collection of plutonic xenoliths from Martinique in order to establish the mode of formation and the conditions in which they were stored, with the aim of establishing a model of the components making up the crust and the sub-volcanic plumbing system beneath the island. We use an extensive dataset to address the following key questions: Where in the crust do the plutonic xenoliths originate from? Do the samples record crystallisation within a closed system, or do they represent a crystal mush undergoing open system processes? To what extent do the plutonic xenoliths represent cumulates versus frozen portions of magma? How does the mineral chemistry vary depending on the evolutionary history? What are the processes which led to the crystallisation of contrasting amphiboles?

There are a vast number of studies focussed on in situ mineral chemistry of volcanic rocks; however, the majority of geochemical and isotopic studies on plutonic rocks have concentrated on whole-rock data. For this study, we focus on the in situ trace element concentrations of mineral phases contained within a range of plutonic xenolith types. Trace element variations (in this case, by laser ablation ICP-MS) in crystal phases provide a means to track processes throughout the magmatic history of the plutonic xenoliths and the conditions in which they were formed. By analysing thin sections directly, the textural relationships of the analysed mineral phases can be assessed, therefore allowing us to compare the formation processes of different plutonic xenolith types and the nature of parental melts.

The formation and evolution of cumulates and/or crystal mushes has been studied for layered intrusions such as Skaergaard (McBirney and Noyes 1979; McKenzie 2011) and Rum (Bédard et al. 1988; Holness 2005; Holness et al. 2007; Leuthold et al. 2014). The generation of cumulates was traditionally thought to involve crystal settling onto the base of the magma chamber and then subsequent evolution of the interstitial liquid through crystal growth and compaction in a closed system (Wager et al. 1960). Solidification likely occurs at magma chamber margins, and therefore exchange of melt to and from the magma chamber may occur. Crystallisation under these conditions is termed in situ crystallisation (McBirney and Noyes, 1979). However, these models are unlikely to work in hydrous systems such as active island arcs (Meurer and Claeson 2002). Cumulate rocks from exposed arc crustal sections (Murray 1972; Greene et al. 2006; Bouilhol et al. 2015; Stuart et al. 2015) and plutonic xenoliths (Smith 2014; Stamper et al. 2014) can also record evidence for open system processes such as multiple magma replenishment episodes or percolating melts. Recently, the evolving liquids interacting with crystal mush and/or cumulate can be traced through the use of in situ trace element concentrations (Meurer and Claeson 2002) and large variations in incompatible trace elements may indicate an open system and an addition of melts into a crystal mush or cumulate pile. Here we investigate the extent of closed and open system processes recorded by plutonic xenoliths.

Amphibole is rarely present in the erupted volcanic products of the Lesser Antilles (with the exception of Montserrat), but it is a very common component in plutonic xenoliths from every island of the Lesser Antilles arc. The trace element signatures of arc lavas suggest that in a water-rich, mid- to lower sub-arc crust, the fractionation of amphibole imparts a control on the differentiation of arc magmas - the amphibole ‘sponge’ model of Davidson et al. (2007). This model is supported by the presence of ultracalcic nepheline-normative melts in the lesser Antilles and other island arcs worldwide, which may be generated by the melting of olivine-clinopyroxene-amphibole cumulates in the lower arc crust (Schiano et al. 2000; Médard et al. 2004). The plutonic xenoliths from Martinique therefore allow us to directly test what the involvement of amphibole and ‘cryptic’ amphibole fractionation (Davidson et al. 2007) has on the petrogenesis of erupted arc magmas and the depths in the crust where these melts are stored and generated. Here, we study amphiboles with contrasting textural relationships and trace element signatures to explore the different magmatic processes involved in their formation.

### Geological setting

The lesser Antilles Arc is located along the eastern margin of the Caribbean plate as the result of the relatively slow (~2 cm/year) westward subduction of the Atlantic oceanic lithosphere. The arc is 750 km long, and to the north it bifurcates into an older arc to the east and the recent arc to the west (Fig. 1). The distinct westward jump occurred at ~7 Ma and has been attributed to the flattening of the subducting slab by the subduction of an aseismic ridge (Bouysse and Westercamp 1990). There is an extensive geochemical variation along the arc, which to a first order is systematic, (Brown et al. 1977; Smith et al. 1980; Turner et al. 1996; Macdonald et al. 2000), although the large geochemical and isotopic variations at each volcanic centre add to the complexity (Bezard et al. 2015). In general, islands in the north (Saba to Montserrat) produce low-K basalts, whereas those to the south (Grenadines and Grenada) produce medium-K picrites and ankaramites (Macdonald et al. 2000) and only the southern islands have mafic magmas with >8 wt% MgO and associated mantle-derived xenoliths (Arculus 1976; Heath et al. 1998). The central islands are typically composed of medium-K basalt or basaltic andesite, although many islands have both low- and medium-K lavas (Macdonald et al. 2000).

Seismic refraction experiments (Boynton et al. 1979) and receiver function analysis (Schlaphorst et al. 2014) reveal a significant variation in the depth of both the inferred MOHO and Conrad discontinuity along the length of the Lesser Antilles volcanic arc. Similar along strike crustal variations have been linked to geochemical composition of the erupted volcanic products, such as in the Izu-Bonin intra-oceanic arc (Kodaira et al. 2007; Tamura et al. 2009). This implies that the crustal structure imparts a control on the petrogenesis of arc lavas. In this study, we are able to directly analyse parts of an active plumbing system, at a potentially diverse range of depths, in order to test the petrogenetic controls of Lesser Antilles arc crust.

Martinique is located in the central arc at the point where the old and current arcs diverge and therefore it displays a complete geologic history of the arc, spanning at least the last 25 Myr (Germa et al. 2011). Distinct volcanic phases make up the old, intermediate and recent volcanic activity in Martinique (Labanieh et al. 2012). The lavas contain, in order of abundance, phenocrysts of: plagioclase, orthopyroxene and clinopyroxene. Amphibole is typically absent and when present (<5 %) it is opacitized (Davidson and Wilson 2011). The lavas from Martinique cover most of the chemical and isotopic variability known in the Lesser Antilles arc (Davidson 1983, 1986; Davidson and Wilson 2011). The large range in isotopic compositions displayed in Martinique lavas is attributed to the incorporation of slab-derived sediment, as well as the addition of sediment melt and fluid via crustal contamination. (Davidson and Wilson 2011). The question then arises as to whether the plutonic counterparts to the lavas also display the same level of geochemical heterogeneity, or whether the processes responsible for the compositional variation occurred in shallow melt-dominant bodies, unrelated to cumulate crystallisation.

Numerous studies have focused on plutonic xenoliths from the Lesser Antilles volcanic arc. Arculus and Wills (1980) provided the first detailed petrological study reporting that the compositions of phases within cumulate xenoliths were distinct from the phenocrysts in associated eruptives. The majority of xenoliths are ad- and heteradcumulates with fewer ortho- and crescumulates. Plagioclase, amphibole, clinopyroxene, orthopyroxene, olivine, magnetite, biotite, ilmenite, quartz and apatite are present in various proportions, and interstitial glass is often present (Arculus and Wills 1980). Significant variation along the arc manifested in the rarity of orthopyroxene and abundance of amphibole in the southern islands, compared with the common presence of two pyroxenes in the northern islands (Arculus and Wills 1980). Plutonic xenoliths from each individual island have distinctive characteristics in terms of mineralogy and petrology (e.g. Arculus and Wills 1980; Kiddle et al. 2010; Stamper et al. 2014; Tollan et al. 2012) which influences the petrology and geochemistry of the juvenile erupted material on each of the islands.

### Analytical techniques

Whole-rock major (SiO_2_, TiO_2_, Al_2_O_3_, Fe_2_O_3_, MnO, MgO, CaO, Na_2_O, K_2_O, P_2_O_5_) and selected trace elements (V, Cr, Rb, Nb, Sr, Y, Zn, Co, Ni, Ba) were analysed by X-ray fluorescence spectrometry using a Siemens SRS 3000 sequential XRF spectrometer at the University of Auckland.

Whole-rock trace element analysis was carried out by solution ICP-MS on a ThermoScientific X-Series 2 ICP-MS at Durham University. W-2, BHVO-1 and AGV-1 standards were used to monitor accuracy and precision (Online Resource 1).

Major element concentrations of minerals were analysed in situ on polished thin sections with Cameca SX100 and JEOL JXA8530F electron microprobes at the University of Bristol. The Cameca SX100 was run with a 20 kV accelerating voltage, a 20 nA beam current and a 1 μm spot size. The JEOL JXA8530F was run with a 15 kV accelerating voltage, a 10 nA beam current and a 1 μm spot size. The instruments were calibrated using synthetic oxide, mineral and metal standards. Typical detection limits are presented in Online Resource 1.

Trace element concentrations of minerals were determined by laser ablation ICP-MS using a New Wave UP193FX laser ablation system coupled to a ThermoScientifc X- Series 2 ICP-MS at Durham University. Analyses were made in situ on the same polished thin sections used for major element analyses. Mineral major element concentrations were determined for each analytical spot by EPMA prior to LA-ICP-MS analysis. This ensures major and trace element concentrations could be coupled and an accurate ^43^Ca (or ^29^Si) value could be used to normalise LA-ICP-MS data. A spot size of 75 μm was used at a laser repetition rate of 5 Hz and a pulse energy of ~5 mJ. Helium was used as the carrier gas. The NIST 612 and NIST 610 glasses were used for calibration and to monitor instrumental drift. BCR-2G and BHVO-2G were used as secondary standards and were analysed during each analytical session (uncertainties presented in Online Resource 1).

## Results

### Plutonic xenolith samples

All studied samples are coarse-grained intrusive rocks and are here termed, collectively, *plutonic xenoliths*. They have been named using the classification scheme of Streckeisen (1976) and are only termed cumulate if the bulk composition, textures and mineral chemistry is consistent with them being a subtractive assemblage. If its cumulate origin cannot be demonstrated through geochemistry, we refer to the rock as a ‘non-cumulate gabbro’, which in this case, represents a magma that has solidified without significant movement of crystals with respect to the host melt. The majority of plutonic xenoliths used in this study were sourced in the north of Martinique, where the most recent phases of volcanism are focussed (Westercamp et al. 1989; Germa et al. 2011). Most xenoliths were sampled ex situ, from riverbeds where they have become gravitationally concentrated. Therefore, samples cannot be directly linked to the deposits in which they were erupted originally. However, the samples used here are inferred to be from Mount Peleé (126–0 ka) and Mount Conil (550–127 ka) (Westercamp et al. 1989; Germa et al. 2011). The Mount Conil complex is made up of andesitic breccias, lava domes and lava flows, the majority of which are now buried beneath the younger explosive deposits of Mount Peleé (Germa et al. 2011), which dominates the north end of the island.

**Fig. 2:**
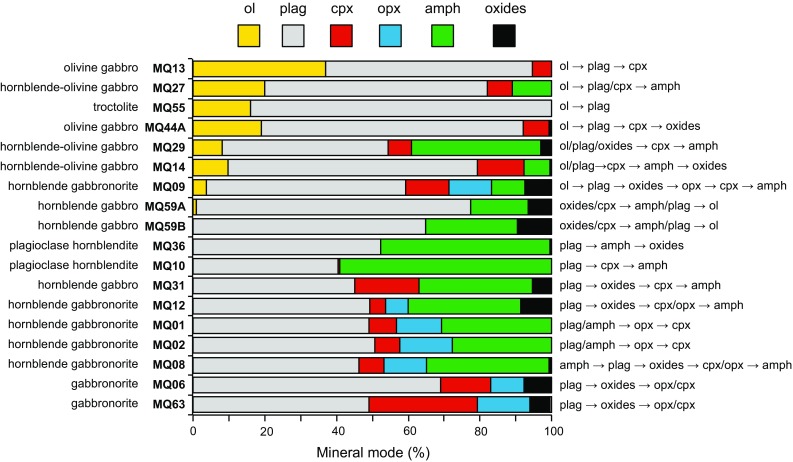
Modal proportions of crystal phases within Martinique plutonic xenoliths (excluding vesiculated glass). Mineral modes were obtained through point counting of thin sections. Samples are listed from *top* to *bottom* by decreasing Fo of olivine followed by An of plagioclase. Names are based on the classification scheme of Streckeisen (1976). The crystallisation sequence (based on textural inspection) of each sample is also given

**Fig. 3:**
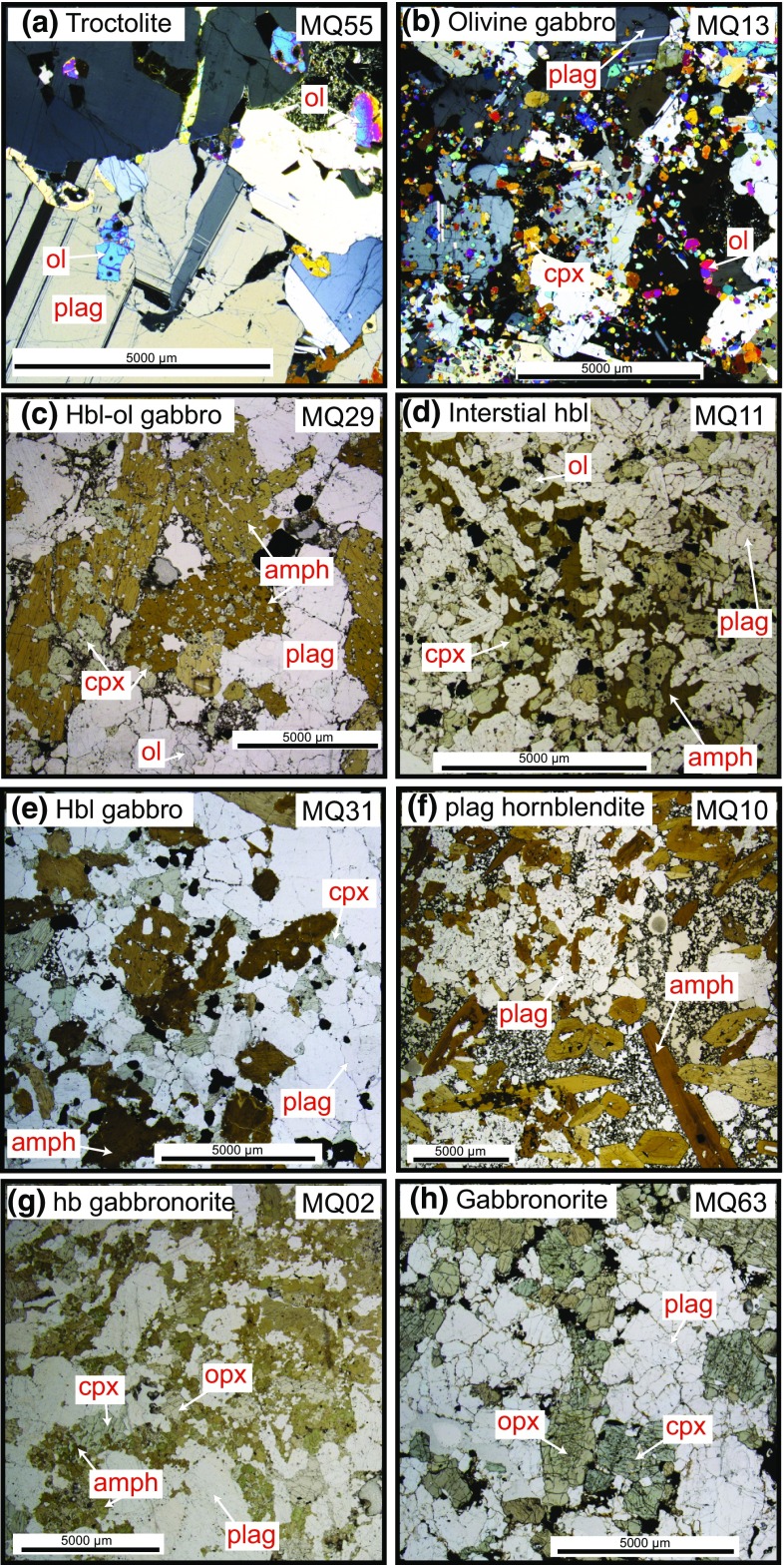
Photomicrographs of plutonic xenolith types displaying characteristic textures. **a** Troctolite with large plagioclase and smaller olivine. Olivine in contact with interstitial glass is zoned. **b** Olivine gabbro with distinctive texture with large plagioclase grains with many small inclusions of olivine. **c** Hornblende–olivine gabbro displaying poikilitic amphibole with clinopyroxene inclusions. **d** Hornblende–olivine gabbro with interstitial amphibole surrounding clinopyroxene. **e** Hornblende gabbro with plagioclase included in clinopyroxene and amphibole. **f** Plagioclase hornblendite with characteristic interstitial glass. **g** Hornblende gabbronorite with poikilitic amphibole with clinopyroxene and orthopyroxene inclusions. **h** Gabbronorite with 120° equilibrium grain boundaries. Both **g** and **h** are termed non-cumulate gabbros (see text)

The relative crystallisation sequence of each plutonic xenolith can be determined through textural inspection and this varies between sample types (Fig. 2). Where present, olivine is always the first phase to crystallise, followed by plagioclase which is ubiquitous and the dominant mineral phase in almost all samples (Figs. 2, 3). In general, clinopyroxene is the next phase to crystallise (co-crystallising with orthopyroxene in gabbronorites). The appearance of spinel varies from the last crystallising phase in olivine gabbros, after plagioclase in gabbronorites and the first crystallising phase in a hornblende gabbro sample. In contrast to the lavas, amphibole is an abundant phase, in addition to clinopyroxene and olivine. Amphibole is either present as an early crystallising phase alongside plagioclase and clinopyroxene or has crystallised at a late stage and is interstitial to the cumulus assemblage. Late-stage, interstitial amphibole appears to be texturally associated with clinopyroxene, and we explore this relationship below, with in situ trace element chemistry. Orthopyroxene is present in a number of clinopyroxene bearing samples.

Many samples display variations in modal layering, textures and degree of interstitial melt present. Adcumulate, heteradcumlate and orthocumulate textures are present within Martinique plutonics and a component of interstitial vesiculated glass is a common feature, giving rise to a disaggregated appearance (Fig. 3). Where in contact with interstitial glass, plagioclase may display a lower An rim, representing post-cumulus reaction with the melt. Melt inclusions are very rare within samples from Martinique. The plutonics can be divided into the following rock types: troctolites, olivine gabbros, hornblende–olivine gabbros, hornblende gabbros, plagioclase hornblendites, hornblende gabbronorites and gabbronorites.

#### Plutonic types

*Troctolites* (ol + plag) are dominated by large plagioclase grains (≤1 cm) with smaller (≤3 mm [majority ≪ 1 mm]) irregular shaped, generally unzoned olivine, which is found both included within plagioclase and along grain boundaries (Fig. 3a). Interstitial vesiculated brown glass is present, and olivine is normally zoned with respect to Fe–Mg where in contact with the glass. Plagioclase is unzoned within troctolite samples.

*Olivine gabbros* (ol + plag + cpx ± spl) have a striking texture dominated by large poikilitic plagioclase grains (≤5 mm) with very small unzoned olivine inclusions (<0.5 mm) (Fig. 3b). Smaller plagioclase grains (<1 mm) are found interstitially together with poikilitic clinopyroxene (<10 %, <2 mm) and minor spinel, which contain both plagioclase and olivine. Plagioclase is unzoned within olivine gabbro samples.

*Hornblende*–*olivine gabbros* (ol + plag + cpx + amph ± opx ± spl) can be subdivided into two groups based on texture and the appearance of amphibole. One type contains large (3 mm– >1 cm) poikilitic amphibole with clinopyroxene inclusions (Fig. 3c). The other type displays an adcumulate texture (120° grain boundaries) with amphibole appearing interstitially and in association with clinopyroxene (Fig. 3d). Grains are <3 mm, and no interstitial glass is found in this type. Plagioclase (≤1 cm) is modally dominant (Fig. 2). Olivine is typically larger (≤3 mm) than in olivine gabbros and often displays weak normal Fe–Mg zoning. Plagioclase is generally unzoned, apart from one sample in which the plagioclase has reacted with the host melt, forming a normally zoned rim. Interstitial glass is present in variable proportions and contains skeletal plagioclase with similar compositions to the reacted plagioclase rims.

*Hornblende gabbros* (plag + cpx + amph ± spl) are characterised by an assemblage dominated by plagioclase and large (3 mm– >1 cm) poikilitic amphibole with both plagioclase and clinopyroxene inclusions. Clinopyroxene (≤3 mm) also contains inclusions of plagioclase (Fig. 3e). Plagioclase is unzoned apart from a number of reacted rims where in contact with interstitial glass.

*Plagioclase hornblendites* (plag + amph ± cpx ± spl) are comprised of roughly equal proportions of large (0.1–1 cm) euhedral amphibole and smaller (<3 mm) plagioclase. An amphibole grain with a clinopyroxene core is found in one sample and scarce spinel is present in another. Interstitial glass (>10 %) is characteristic of the plagioclase hornblendite samples (Fig. 2f). Skeletal plagioclase microlites are found within the vesiculated glass. Plagioclase is unzoned apart from a number of reacted rims where in contact with interstitial glass.

*Hornblende gabbronorites* (plag + amph + cpx + opx ± ol ± spl) are dominated by plagioclase (normally zoned in a number of samples), and orthopyroxene and clinopyroxene are present, the former in a greater proportion. Amphibole appears before or together with plagioclase in the crystallisation sequence. Larger amphibole commonly contains inclusions of clinopyroxene and orthopyroxene which may be breaking down (Fig. 3g). Portions of hornblende gabbronorite samples have a granoblastic texture. Bent plagioclase twins are present in a number of samples indicating some deformation. A number of these samples do not appear to have a cumulate origin.

*Gabbronorites* (plag + cpx + opx ± spl ± apatite) are dominated by normally zoned plagioclase and are typified by the absence (or trace amounts) of amphibole (Fig. 3h). Both clinopyroxene and orthopyroxene are present, the former in a greater proportion. These samples contain both interstitial magnetite and ilmenite, which crystallised late, and large (1-mm length) needle-shaped apatite is also present. Pyroxene exsolution lamellae are present in one non-cumulate gabbro sample. Bent plagioclase twins are present in a number of samples indicating some deformation. These samples do not appear to have a cumulate origin.

### Lava and plutonic xenolith whole-rock chemistry

**Fig. 4:**
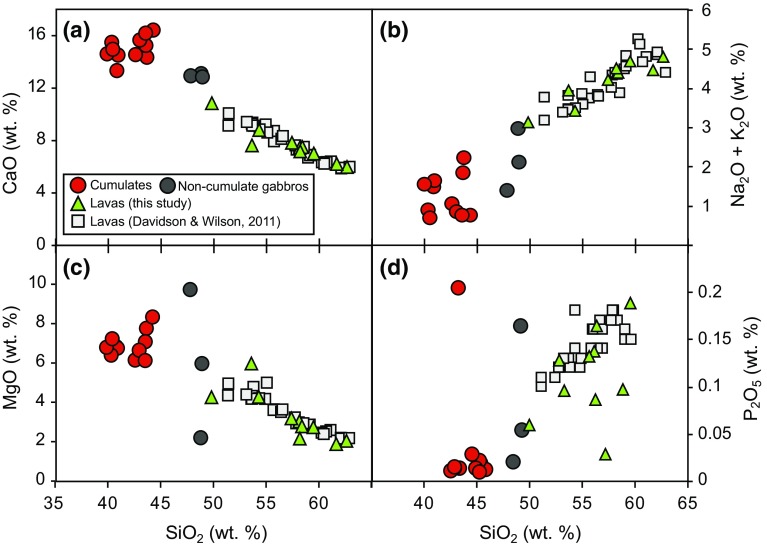
Whole-rock major element chemistry of Martinique cumulates and lavas from this study and Davidson and Wilson (2011)

**Fig. 5:**
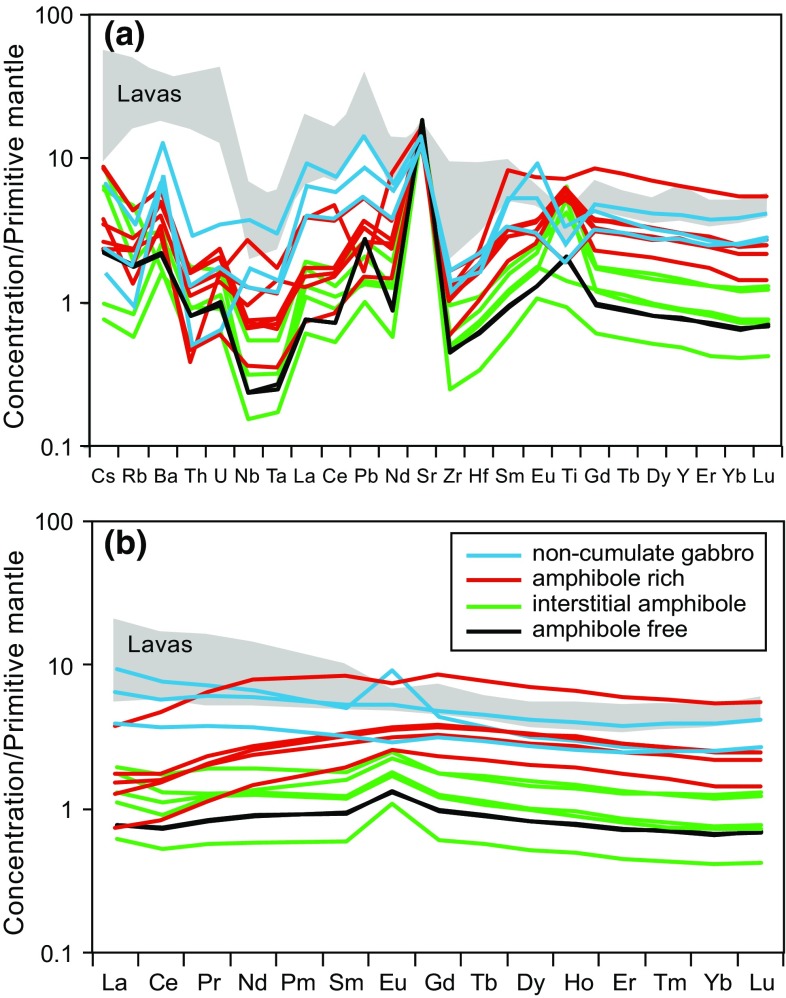
**a** Extended whole-rock trace element spidergram and **b** REE diagram of Martinique lavas (data from Davidson and Wilson (2011) and this study) and plutonic xenoliths, normalised to primitive mantle (Palme and O’Neill 2003)

Ten crystal-rich lavas and 14 plutonic xenoliths were analysed for whole-rock major and trace element chemistry. The Martinique lavas overlap the compositions of Mt Peleé lavas analysed by Davidson and Wilson (2011). Lavas analysed in this study range from Medium-K basalts to andesites (50–63 wt% SiO_2_). CaO ranges from 6 to 11 wt%, Na_2_O + K_2_O ranges from 3.2 to 4.8 wt% and P_2_O_5_ from 0.03 to 0.2 wt% (Fig. 4). The lavas define typical fractionation trends with decreasing CaO and MgO and increasing total alkalis with increasing SiO_2_ (Fig. 4). P_2_O_5_ positively trends with SiO_2_, but with some scatter in lavas, and there is no fractionation peak to indicate apatite saturation (Fig. 4). Lavas display typical island arc trace element patterns with enrichment in LILE (Fig. 5a). Chondrite-normalised REE patterns have a concave-up shape, consistent with the removal of amphibole, which is relatively enriched in MREE over HREE (concave-down REE pattern; Fig. 5b). Lava samples display greater enrichments in incompatible elements (Cs, U, Th, Pb, Rb and Ba) than their plutonic counterparts (Fig. 5a).

The whole-rock chemistry of the plutonic xenoliths is a direct reflection of their crystal assemblage and the majority of compositions of the analysed samples are consistent with a cumulate origin. However, three of the analysed gabbroic xenoliths have chemistries closer to lava compositions and therefore we have termed these *non*-*cumulate gabbros* (Fig. 4). The plutonic xenoliths lie outside the field defined by the lavas on major element plots and do not follow a liquid line of descent (Fig. 4). Plutonic xenoliths have higher CaO (>12 wt%) and MgO (>5 wt%) and lower Na_2_O + K_2_O (<3 wt%) and P_2_O_5_ (<0.05 wt%) to lavas (Fig. 4). In contrast to lavas, trace element spidergrams reveal plutonic xenoliths have a stronger positive Sr anomaly, and have positive Ti anomalies (Fig. 5a). Amphibole-rich xenoliths and samples in which amphibole has crystallised early have concave-down chondrite-normalised REE patterns and lack Eu anomalies (Fig. 5b). Xenoliths which are amphibole free, or in which amphibole has crystallised late, have positive Eu anomalies, reflecting the high modal proportions of plagioclase (Fig. 2).

**Fig. 6:**
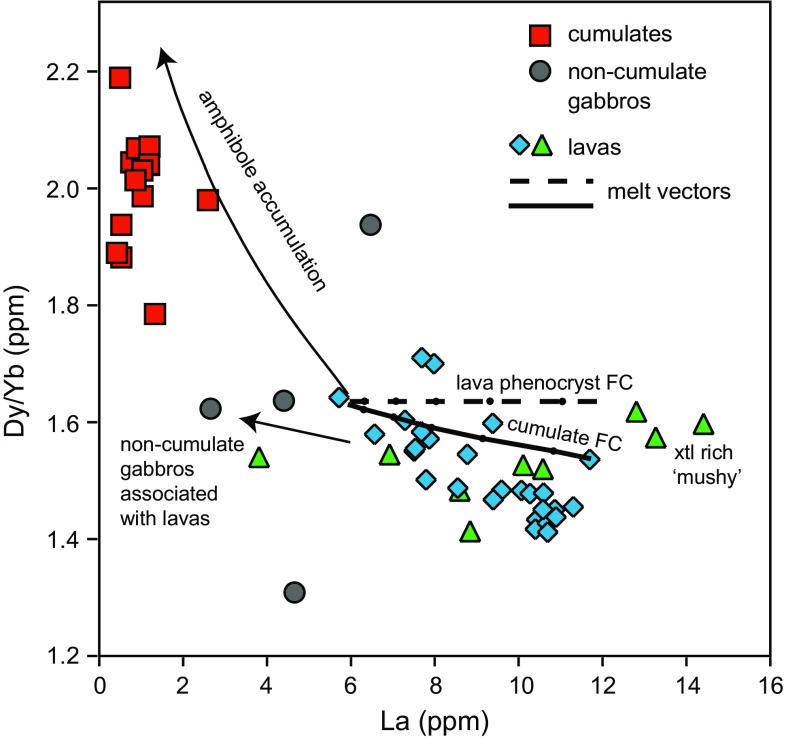
Whole-rock La versus Dy/Yb of Martinique lavas and plutonic xenoliths. *Solid* and *dashed lines* are modelled melt vectors from the fractional crystallisation of either the lava phenocryst or the cumulate assemblage (after Davidson and Wilson 2011)

The amphibole-bearing plutonic xenoliths have higher Dy/Yb ratios (~1.8–2.2) and lower La (0–7 ppm) than lavas (Dy/Yb ~1.4–1.7, La 6–14 ppm) (Fig. 6). However, a subset of gabbroic (non-cumulate) samples have similar Dy/Yb to lavas (~1.3–1.9). The non-cumulate gabbros are either hornblende gabbronorites or gabbronorites. Minerals from these samples also have distinctive chemistries (discussed below), suggestive of a different origin to the cumulate plutonic xenolith samples.

### Mineral major and trace element chemistry

**Fig. 7:**
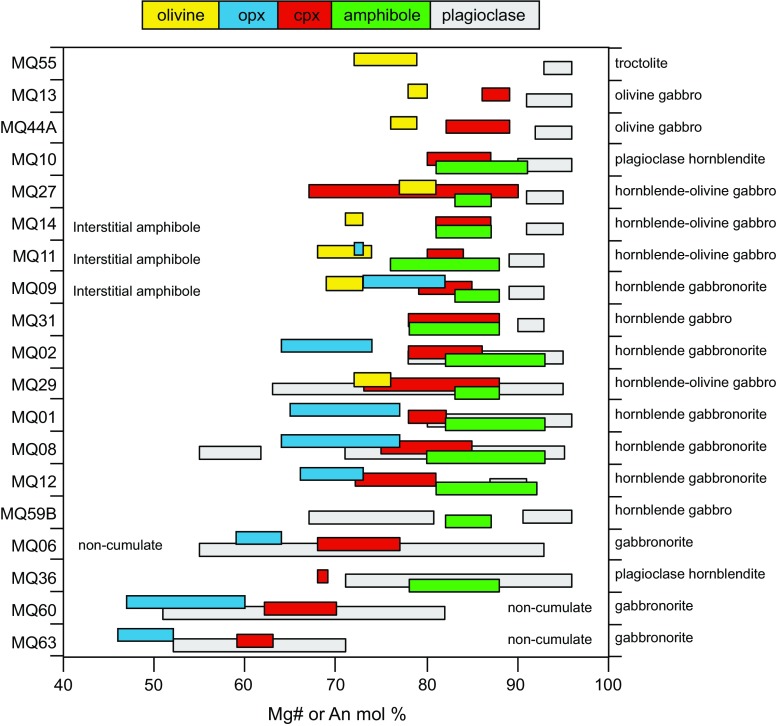
Summary of phase compositions for the range of studied plutonic xenolith types: Mg# (100 Mg/(Mg + Fe^2+^) of olivine, orthopyroxene, clinopyroxene, amphibole, and An (mol%) of plagioclase. Samples are ordered based on the mean clinopyroxene Mg#

Here, the in situ major and trace element concentrations of olivine, plagioclase, amphibole, clinopyroxene, orthopyroxene and spinel are summarised. The range in Mg number of olivine, opx, cpx and amphibole, and the An (mol%) of plagioclase are summarised in Fig. 7. The full dataset is presented in Online Resource 1.

#### Olivine

**Fig. 8:**
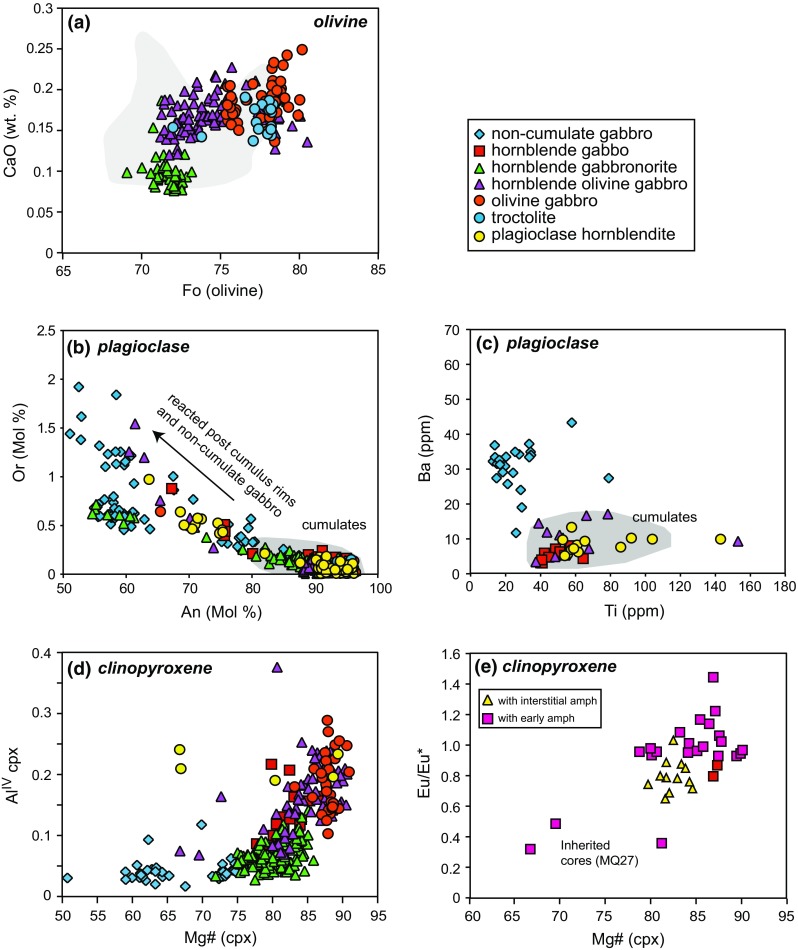
**a** Olivine Fo versus CaO (wt%) from different plutonic xenolith types. *Grey shaded* area represents range covered by Grenada cumulates (Stamper et al. 2014). **b** Major and **c** trace element chemistry of plagioclase from different plutonic xenolith types. *Grey shaded* areas represent the compositional range covered by plutonic xenoliths with a cumulate origin. **d** Major element variations of clinopyroxene from different plutonic xenolith types. **e** Mg# versus Eu/Eu* of clinopyroxene from samples with either early or interstitial amphibole

Olivine is present in <50 % of studied samples and has a relatively narrow range in composition (Fo_81–68_) (Fig. 8). The olivine is pristine with no signs of alteration to iddingsite or serpentine, in marked contrast to Grenada (Stamper et al. 2014). CaO contents are <0.25 wt% and Ni contents are low (40–750 ppm) (Fig. 8). Ni contents are similar to those from St. Vincent (40–720 ppm; Tollan et al. 2012), but significantly lower than cumulates from Grenada which range from 0–0.3 wt% (Stamper et al. 2014). The CaO content varies with sample type from an average of 0.1 wt% in hornblende gabbronorites to 0.2 wt% in olivine gabbros (Fig. 8a). The majority of olivine is unzoned; however, when in contact with interstitial glass, a number of grains display normal zoning (lower Fo rims) reflecting reaction with interstitial melt. Transition metals are the only group of trace elements with significant abundances (3200–7600 ppm Mn, 136–390 ppm Zn, 226–336 ppm Co). Mn and Zn concentrations increase with decreasing Fo, but no clear trend is shown by Co.

#### Plagioclase

Plagioclase is modally dominant in all studied samples (Fig. 2) and is, in general, anorthite-rich (An_96–70_; Fig. 7). Those analyses of plagioclase <An_80_ are from plagioclase rims, which are often in contact with interstitial glass, and therefore represent a reacted rim. In the majority of samples, the interiors of plagioclase are unzoned, apart from in gabbronorites in which it is oscillatory zoned. Oscillatory zoned plagioclase is also present within Martinique lavas (Pichavant et al. 2002; Davidson and Wilson 2011). The range of plagioclase compositions within the gabbronorite samples is greater and more sodic (An_93–51_) and covers a similar range to zoned plagioclase within Martinique lavas which have calcic cores An_85–90_ and sodic rims An_50–65_ (Pichavant et al. 2002; Fig. 8b).

Plagioclase displays typical REE characteristics of a relative LREE/HREE enrichment and a positive Eu anomaly. Sr concentrations are higher in an olivine gabbro sample (900–1300 ppm) compared with a range of 300–550 ppm in other sample types. Sr and Ba concentrations do not show systematic variations with An. Ba is enriched in plagioclase from gabbronorites (11–43 ppm) compared with other sample types (3–20 ppm) (Fig. 8c). Ti (13–154 ppm) and Mn concentrations (18–100 ppm) are highest in olivine gabbros and lowest within gabbronorites in which spinel has crystallised before plagioclase (Fig. 8c).

#### Clinopyroxene

Clinopyroxene is present in all samples, with the exception of one troctolite. Compositions are Ca-, Al- and Fe^3+/^ΣFe-rich diopsides with Mg# 90–67. Fe^3+/^ΣFe, determined through stoichiometry, ranges from 0.0–0.6 and decreases with decreasing Mg#. Clinopyroxene from olivine gabbros and hornblende–olivine gabbros the most enriched in Fe^3+^. Gabbronorite samples have lower Mg compositions (Mg# 77–59) (Fig. 8d). Grains are either unzoned or have patchy sector zoning. There is a large variation in the concentration of Al_2_O_3_; clinopyroxene from olivine gabbros and hornblende gabbros are enriched (2.5–9.0 wt% Al_2_O_3_) compared with those from gabbronorites (1.0–4.3 wt% Al_2_O_3_). This is reflected in Al^IV^, with a range in olivine gabbros and hornblende gabbros of Al^IV^ 0.1–0.3, compared to Al^IV^ <0.1 in gabbronorites (Fig. 8d).

The trace element concentrations of clinopyroxene vary with sample type and whether amphibole crystallised early or late in the sequence. Clinopyroxene associated with interstitial amphibole have small Eu/Eu* ~0.85, whereas clinopyroxene associated with early crystallising amphibole does not display an Eu anomaly. Within one olivine gabbro sample (MQ27), a number of clinopyroxene cores have a strong negative Eu/Eu* of ~0.4 with Mg# 67–82; however, the rims of these grains do not have a Eu anomaly and have higher Mg# (80–90; Fig. 8e).

**Fig. 9:**
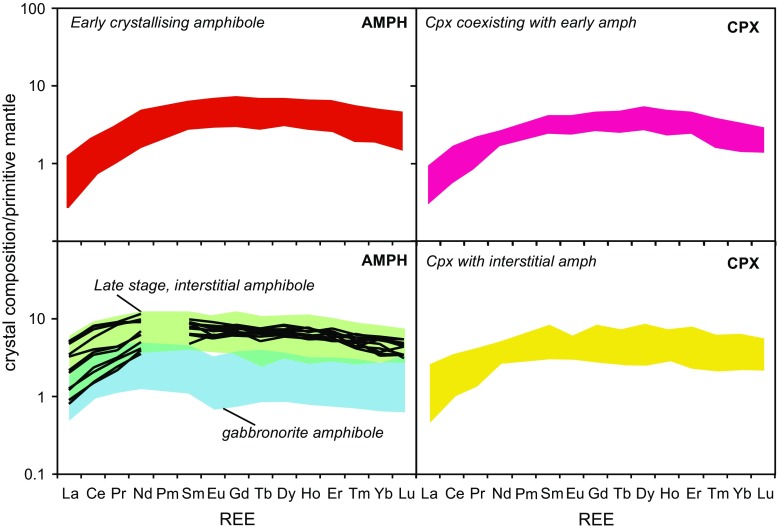
REE patterns of amphibole and clinopyroxene types normalised to primitive mantle values (Palme and O’Neill 2003). *Black lines* within the *shaded area* of late-stage, interstitial amphibole are the REE in one sample to show the variation in LREE

Clinopyroxene displays a large range in incompatible trace elements, highlighted by the range shown by Hf and Zr (Online Resource 2). Grains from samples containing early crystallising amphibole have lower concentrations of incompatible trace elements than grains associated with late crystallising amphibole. The full range in clinopyroxene Hf and Zr from each type is covered by a single plutonic xenolith sample. All clinopyroxene has enrichments in MREE and HREE compared to LREE (Fig. 9). In samples with early crystallising amphibole, the clinopyroxene REE patterns have a humped profile compared to a flatter profile in samples containing late crystallising amphibole (Fig. 9). There is a greater variation in the LREE concentrations of clinopyroxene associated with late amphibole (La/Yb of 0.17–1.56) compared to grains in samples in which amphibole crystallised early (La/Yb of 0.13–0.7).

#### Orthopyroxene

Orthopyroxene is present in ~50 % of the studied samples and only occurs in assemblages in which clinopyroxene is present. Compositions range from Mg# 64–82 (Fig. 7) and En_75–60_ (Online Resource 3) in amphibole-bearing cumulate samples. Orthopyroxene in amphibole free, and non-cumulate gabbros are less magnesian (Mg# 64–46 and En_60–44_). Exsolution lamelle contained in one sample is more calcic (>Wo_4_). Nickle concentrations are lower and Ti concentrations are higher in olivine-bearing gabbros (Ni 10–30 ppm, Ti 840–1670 ppm) compared with olivine free gabbros (Ni 95–202 ppm, Ti 440–1030 ppm; Online Resource 3). Zinc (290–400 ppm) and V (45–160 ppm) are consistent between sample types and do not vary systematically with major element concentrations.

#### Amphibole

**Fig. 10:**
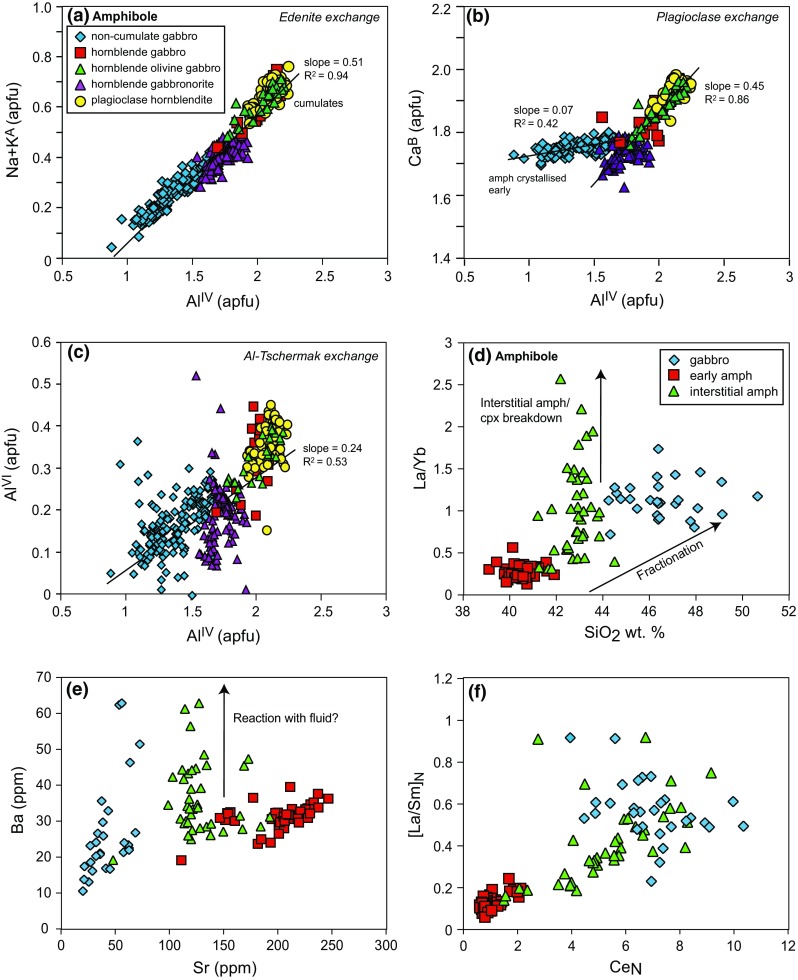
Major and trace element variations of amphibole. Structural components (Leake et al. 1997) are used to show variation in major elements and **a** edenite, **b** plagioclase and **c** Al-Tschermak exchange. The trace element variations of early, interstitial and non-cumulate gabbro amphibole (**d**–**f**) highlight enrichments in LREE and fluid mobile elements within interstitial amphibole

Amphibole compositions based on Leake et al. (2003) vary between sample types; magnesio-hastingsite is the dominant type in hornblende–olivine gabbros and hornblende gabbros; tschermakite-pargasite is common in hornblende gabbonorites and magnesio-hornblende in gabbronorites. Compositions cover a narrow range in regard to Mg# (93–78; Fig. 7), but a large range in Al (Al^IV^ 0.88–2.25) with euhedral, early crystallising amphibole more aluminium-rich than interstitial and poikilitic amphibole (Fig. 10). Na + K^A^ ranges from 0.04–0.76 and positively correlates (slope = 0.51) with Al^IV^ indicating temperature-sensitive edenite exchange is significant (Fig. 10a). Ca^B^ versus Al^IV^ indicates that plagioclase exchange is significant in amphibole from the majority of plutonic xenoliths (Fig. 10b). However, plagioclase exchange is insignificant in amphiboles from hornblende gabbronorite samples, in which amphibole crystallised before plagioclase. Al^IV^ versus Al^VI^ indicates the extent of the pressure sensitive Al-Tschermack substitution (Fig. 10c). There is no trend in Al^IV^ versus Al^VI^ within each xenolith type, with the exception of non-cumulate gabbro. However, differences in Al^IV^ and Al^VI^ between xenolith types may suggest a relative shift in crystallisation pressures.

Within Martinique plutonic xenoliths, late-stage, interstitial amphibole appears to be texturally associated with clinopyroxene, and this observation is reflected in the trace element chemistry of amphibole. Like clinopyroxene, amphibole shows a large variation in incompatible trace elements, highlighted by the range shown by Hf and Zr, which span an order of magnitude (Online Resource 2). Early amphibole has lower concentrations of incompatible trace elements (Hf, Zr, Zn) compared with interstitial amphibole, and the ratios between incompatible trace elements are not consistent across amphibole types (i.e. different slopes on Hf versus Zr plot [Online Resource 2]). Interstitial amphibole is more enriched and has a larger range in LREE (and Ba) compared with early amphibole (Fig. 10). This is particularly evident in amphibole from an olivine gabbro sample (MQ14), which displays a fivefold variation in La (Fig. 9). Amphibole that has crystallised early displays concave-down or ‘humped’ REE profile, with a MREE/HREE enrichment and a significant LREE depletion. In contrast, interstitial amphibole has flatter REE profiles, with less depletion in LREE and only moderate MREE/HREE fractionation (Fig. 12). It is important to note that neither the early or late crystallising amphibole display a negative Eu anomaly. The amphibole REE profiles have very similar shape to those of the associated clinopyroxene within sample types (Fig. 9). This suggests the amphibole may have formed from the breakdown of clinopyroxene, a reaction that is explored further within the discussion section.

#### Spinel

Spinel is present in >50 % of studied samples. There are two distinctive spinel compositions present, depending on the presence of coexisting ilmenite (Online Resource 4). Coexisting magnetite and ilmenite are present within the three non-cumulate gabbronorites, and these have a higher TiO_2_, lower Al_2_O_3_ magnetite present. Within the amphibole-bearing cumulate gabbros, only a single-phase, an Al-rich magnetite is present in each sample.

## Discussion

### Mineral chemistry

There is a marked contrast between the composition of plagioclase (high anorthite) and coexisting (low Fo) olivine within Martinique plutonic xenoliths, which is a common feature of plutonic rocks from both the Lesser Antilles and global arc settings (Online Resource 5). Generally, this is attributed to high H_2_O contents, which suppresses plagioclase crystallization until the fractionation of mafic phases has substantially depleted the melt in MgO. However, in plutonic xenoliths from Martinique, plagioclase is a ubiquitous phase and commonly crystallises before clinopyroxene and amphibole. In addition, the coexisting compositions have not been reproduced by experimental studies performed on appropriate melt compositions (Sisson and Grove 1993a; Pichavant and Macdonald 2007). A two-stage polybaric differentiation could account for the observed plagioclase and olivine compositions, as suggested for the evolution of St. Vincent magmas (Melekhova et al. 2015). In this case, the olivine-bearing samples have crystallised from differentiated basaltic andesite magmas, rather than as residual assemblages from deep crustal source regions, where the melts were generated (Melekhova et al. 2015). Olivines from Martinique have relatively low Fo and Ni contents, providing evidence of crystallisation from a melt that has undergone prior olivine fractionation and is consistent with early differentiation in the deep crust.

There is a clear distinction in the major and trace element chemistry of crystal phases between plutonic xenolith types. It is possible to distinguish whether a sample is of cumulate origin by using the compositions of crystal phases, and these support the textural evidence and whole-rock compositions. In general, non-cumulate gabbros contain crystals with a larger range and more evolved compositions than cumulate equivalents. Non-cumulate gabbros include both gabbronorite and hornblende gabbronorite assemblages which represent ‘frozen’ portions of a melt. Plagioclase within non-cumulate gabbros are oscillatory zoned and cover a similar range in compositions to phenocrysts within erupted lavas (Pichavant et al. 2002). There is a distinctive group of low An and Or (mol%) plagioclase compositions which lie off the main trend of the cumulate plagioclase (Fig. 8). This group corresponds to plagioclase rims within a number of non-cumulate gabbro samples and likely represents crystallisation after post-cumulus interaction with their host lavas. Similarly contrasting chemistries between cumulate and non-cumulate gabbros are observed in both clinopyroxene and orthopyroxne (Fig. 8 and Online Resource 3).

### Intensive variables and the origin of plutonic xenoliths

Determining the storage conditions (*P*–*T*–H_2_O–ƒO_2_) under which Martinique plutonic xenoliths were formed is essential in understanding the sub-volcanic system beneath Martinique and the evolution of Martinique eruption products. However, constraining reliable estimates of Martinique storage conditions remains challenging for the available plutonic assemblages, and the number of techniques that can be used is limited. Here we use a combination of geothermometers (Putirka 2016; Ridolfi and Renzulli 2012; Holland and Blundy 1994; Ghiorso and Evans 2008) and compare the results with the run conditions of appropriate experimental studies (approach of Stamper et al. 2014; Table 1).

**Table 1:** Comparison of appropriate experimental studies which return assemblages and compositions close to that of the natural plutonic xenolith samples

	Experiment	Compositions		Temperature	Pressure	H_2_O
Troctolite/olivine gabbro	Sisson and Grove (1993b)—Low Mg–high Al basalt	Olivine Fo 72–80	Cpx ~0.1 Al^IV^	1020–1082 °C	0.1 GPa	Saturated
	Pichavant and MacDonald (2007)—High Al basalt	Olivine Fo 75–82	Cpx ~0.2 Al^IV^	1050–1150 °C	0.4 GPa	1.7–5.9 wt%
Plagioclase hornblendite	Pichavant et al. (2002)—Basaltic andesite	Plag An 75–90	Hbl ~1.7 Al^IV^	945–949 °C	0.4 GPa	8.2 wt%
Hornblende gabbro	Pichavant et al. (2002)—Basaltic andesite	Olivine Fo 70	Hbl ~1.8 Al^IV^	1000 °C	0.4 GPa	6.8 wt%
		Plag An 85	Cpx ~0.15 Al^IV^			
	Sisson and Grove (1993b)—High Al basalt	Olivine Fo 77	Hbl ~1.8 Al^IV^	965 °C	0.2 GPa	Saturated
		Plag An 85	Cpx ~0.23 Al^IV^			
	Martel et al. (1999)—Andesite	Plag An 82	Hbl ~1.45 Al^IV^	930 °C	0.2 GPa	6.9 wt%
			Cpx ~0.08 A1^IV^			
(Hornblende) gabbronorite	Pichavant et al. (2002)—Basaltic Andesite	Plag An 62–71	Cpx En 35–54	950–1016 °C	0.4 GPa	3.9–6.9 wt%
			Opx En 54–82			
	Melekhova et al. (2015)—High MgO basalt	Plag An 47–81	Cpx En 44–46	950–1230 °C	0.7–1 GPa	0.6–2.3 wt%
		Hbl 1.75–2.1 Al^IV^	Opx En 77–86			

Experimental studies were chosen if their starting compositions fall on the liquid line of descent, if they reproduce the plutonic types represented in Martinique, and if the phase compositions are close to that of the natural samples (Online Resource 6; Table 1). If experimental crystal compositions reproduce those from natural assemblages, the temperature, pressure and water content can then be inferred from the experimental run conditions. Experiments by Sisson and Grove (1993a) and Pichavant and MacDonald (2007) suggest shallow olivine gabbro crystallisation (0.1–0.4 GPa) at 1020–1150 °C and high water contents (saturated and 1.7–5.9 wt%, respectively). Pichavant et al. (2002) experiments suggest plagioclase hornblendite crystallisation at 0.4 GPa, 945–949 °C and high water contents (8.2 wt%). Sisson and Grove (1993a) and Pichavant et al. (2002) experiments suggest hornblende gabbro crystallisation at 0.2–0.4 GPa, 965–1000 °C and high water contents (saturated and 6.8 wt%, respectively). We therefore infer a common origin to plagioclase hornblendite and hornblende gabbro assemblages. Experiments by Pichavant et al. (2002) and suggest gabbronorite crystallisation at 0.4 GPa, 950–1016 °C and water contents from 3.9–6.9 wt% H_2_O. Experiments by Melekhova et al. (2015) were run at higher pressures (0.7–1 GPa) and lower water contents (0.6–2.3 wt%) and the composition of phases were not as close to natural samples as Pichavant et al. (2002) (Online Resource 6).

**Fig. 11:**
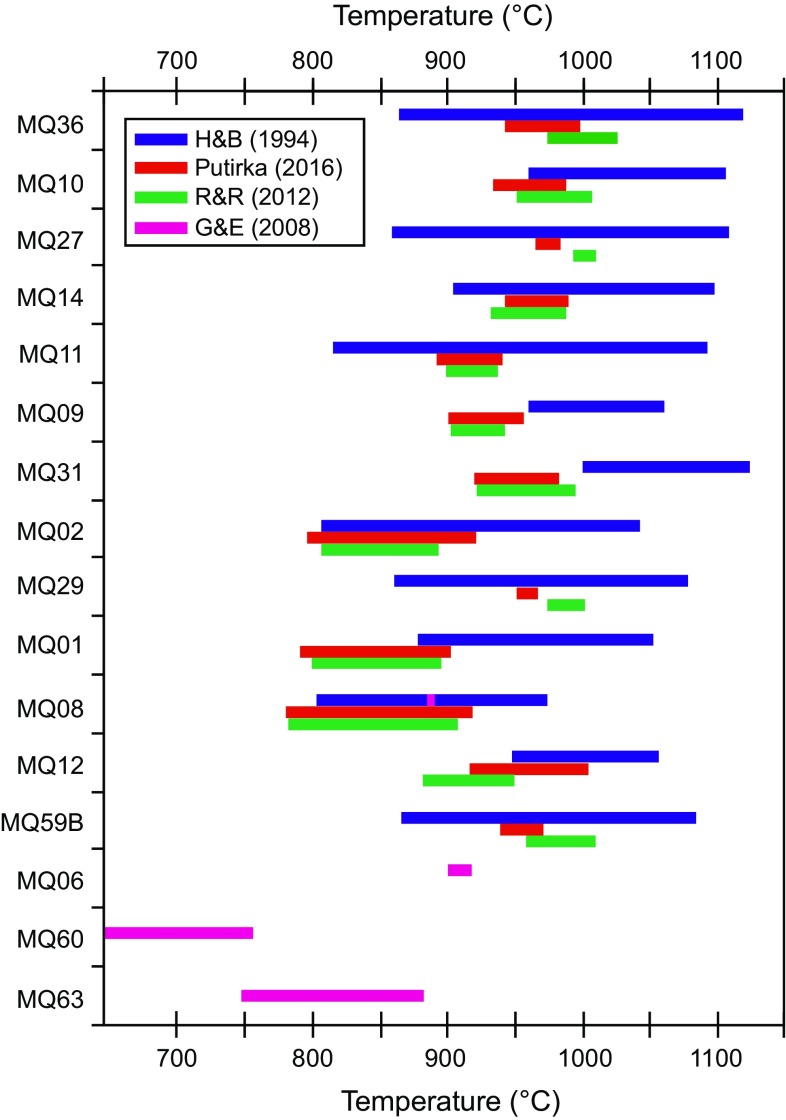
Comparison of temperature estimates using the hornblende–plagioclase model of Holland and Blundy (1994) and the amphibole only models of Ridolfi and Renzulli (2012) and Putirka (2016). In non-cumulate gabbro samples, which contained coexisting magnetite and ilmenite, the Fe–Ti oxide model of Ghiorso and Evans (2008) was used

Model temperature and oxygen fugacity estimates were made using amphibole only geothermometer of Ridolfi and Renzulli (2012) and Putirka (2016). Within Martinique xenoliths, interstitial amphibole is not in textural equilibrium with the cumulate phases, and there is no associated interstitial melt available to test for chemical equilibrium. It has also been shown that H_2_O-rich magmas, which will be relatively Al_2_O_3_-rich, will overestimate crystallisation temperatures (Erdmann et al. 2014). Therefore, amphibole only model temperatures need to be used with caution. However, the model temperatures in amphibole-bearing samples are within the range of those inferred from experimental studies. Amphiboles in cumulates using Ridolfi and Renzulli (2012) return model temperatures of 880–1020 °C, but are lower within non-cumulate gabbros 780–920 °C (Online Resource 7; Fig. 11). Oxygen fugacity estimates using Ridolfi and Renzulli (2012) are 0.6–3.4 ΔNNO and vary between samples (Online Resource 7). Oxygen fugacity estimates of Ghiorso and Evans (2008) of gabbronorite samples (both magnetite and ilmenite present) are −0.2 to 0.8 ΔNNO. Model temperatures using the pressure independent model (Eq. 5) of Putirka (2016) cover a range of 890–1005 °C in cumulates, and 800–908 °C in non-cumulate gabbros and are very similar to those of Ridolfi and Renzulli (2012) (Fig. 11). Temperatures were also calculated using the hornblende-plagioclase model of Holland and Blundy (1994). To increase the likelihood of equilibrium between coexisting hornblende and plagioclase, only the compositions of crystal rims were used to calculate temperatures. The range in temperatures from each sample (100–255 °C) is larger than the range of those calculated from the Ridolfi and Renzulli (2012) and Putirka (2016) models (Online Resource 6; Fig. 11). Holland and Blundy (1994) model temperatures are also higher, with median values close to the hottest of Ridolfi and Renzulli (2012) (Fig. 11). The increased range of temperatures is likely a consequence of disequilibrium between plagioclase and amphibole, particularly in samples with interstitial (late-stage) amphibole (e.g. samples MQ11 and MQ14; Fig. 11). Magnetite and ilmenite are only present together within four of the plutonic xenoliths, and these are classed as non-cumulate gabbros. Where present, temperatures can be estimated using the Fe–Ti oxide model of Ghiorso and Evans (2008). Model temperatures are lower when compared with the other methods (646–909 °C; Fig. 11). This may be a reflection of different storage conditions and the formation of non-cumulate gabbros from more evolved melts.

Amphibole compositions have been shown to have a strong pressure dependence (e.g. Niida and Green 1999; Mandler and Grove 2016). The relative pressure changes between xenolith types are explored using the pressure sensitive Al-Tschermack substitution, shown by Al^IV^ versus Al^VI^ (Fig. 10c). There is no trend in Al^IV^ versus Al^VI^ within each xenolith type, with the exception of non-cumulate gabbro, indicating limited pressure control. This is particularly evident in hornblende gabbronorites, where Al^VI^ shows a large range with little/no corresponding change in Al^IV^ and therefore are likely to be controlled by variations in melt chemistry. However, differences in Al^IV^ and Al^VI^ between xenolith types suggest a relative shift in crystallisation pressures between non-cumulate gabbro and hornblende gabbronorite at lower pressures, and hornblende gabbro, hornblende–olivine gabbro and plagioclase hornblendite at higher pressures (Fig. 10c). Pressure estimates using the amphibole only barometers of Putirka (2016), Ridolfi et al. (2010) and Ridolfi and Renzulli (2012) have been shown to have very large uncertainties (Erdmann et al. 2014; Putirka 2016) and were therefore not calculated for this study.

The comparison to experimental studies suggests that all Martinique assemblages can be produced under high water contents and pressures ≤0.4 GPa corresponding to depths of ≤15 km. Therefore, all plutonic xenoliths from Martinique are samples from mid-upper crustal storage regions and high water contents play an important role in the petrogenesis of magmas. The high anorthite content in plagioclase, high wollastonite content in clinopyroxene, and amphibole compositions indicate crystallisation from a hydrous magma (Gaetani et al. 1993; Sisson and Grove 1993a; Claeson and Meurer 2004). The presence of amphibole within Martinique plutonic xenoliths suggests that mid-upper crystal mushes can act as a sponge, storing and supplying the final erupted melts with water. Davidson and Wilson (2011) apply the geohygrometer of Pichavant and Macdonald (2007) to Martinique lavas, which gives water contents of 3.1–4.5 wt% H_2_O (assuming a temperature of crystallisation of 1050 °C). Such H_2_O contents are consistent with extensive amphibole crystallisation in deep crustal magma reservoirs (Davidson and Wilson 2011), such as the storage regions we directly analyse in this study.

No plagioclase-free assemblages were sampled from Martinique, and there is no evidence that any Martinique plutonic xenoliths have an origin in the lower crust. In hydrous conditions at greater pressures and depths, plagioclase is likely to be absent (Melekhova et al. 2015). Plagioclase-free assemblages have been sampled on Grenada (Stamper et al. 2014) and St. Vincent (Tollan et al. 2012) to the south of the Lesser Antilles arc and plagioclase appears later in the crystallisation sequence in plutonic xenoliths from Grenada. These distinctions may reflect a different crustal structure beneath the islands (Boynton et al. 1979), coupled with a contrasting polybaric petrogenesis.

### Amphibole and reactive melt flow

In contrast to Grenada (Stamper et al. 2014), plutonic xenoliths from Martinique do not have a consistent crystallisation sequence. This is particularly evident in the early or late appearance of amphibole, which are both texturally and compositionally distinct. A variable crystallisation sequence may suggest that the plutonic xenoliths from Martinique are formed from more than one parental melt with distinct evolutionary history and H_2_O content, or they represent the same melt at a different stage of evolution. Amphibole trace element chemistry, together with textural evidence, provides evidence that multiple melts were involved in the petrogenesis of Martinique plutonic xenoliths. Early crystallising (equant) amphibole is more aluminium rich (both Al^IV^ and Al^VI^) and has lower concentrations of incompatible trace elements (Online Resource 2), LREE and Ba than late (interstitial and poikilitic) amphibole (Fig. 10). Melt SiO_2_ (wt%) in equilibrium with early and interstitial amphibole was estimated using formulations from Putirka (2016). Early amphibole returned melts with 50.3–52.8 wt% SiO_2_ compared with more evolved compositions of 58.8–64.6 wt% SiO_2_ from interstitial amphibole (Online Resource 7). Predicted melt compositions of ~70 wt% SiO_2_, and the similarity of mineral chemistry from non-cumulate gabbros and lavas suggests that the non-cumulate gabbros are associated with the final erupted magmas.

The question then arises as to whether the late-stage amphibole crystallised in a closed system from the residual melt after crystallisation of the other phases (e.g. McBirney and Noyes 1979; Morse 1996), or in an open system involving the input of percolating reactive melts or liquids (Reiners 1998; Coogan et al. 2000, 2001; Meurer and Claeson 2002; Leuthold et al. 2014; Smith 2014; Bouilhol et al. 2015; Stuart et al. 2016). It has been shown that the products of reactive liquid flow will differ from one produced by simple crystallisation along a liquid line of descent (Reiners 1998) and the addition of low degree melts cause large variations in incompatible trace elements. In Martinique samples, there is an order of magnitude variation in incompatible elements (Hf and Zr) in amphibole and clinopyroxene (Online Resource 2) and a fivefold variation in La of amphibole is observed within one sample (Fig. 9). Trace element concentrations of interstitial amphibole do not follow the trend predicted if fractional crystallisation controlled the melt chemistry during amphibole crystallisation (Fig. 10d). This suggests that there has been some addition of melt into the system. This evidence is supported by the lack of a negative Eu anomaly in interstitial amphibole. If interstitial amphibole crystallised from a residual melt after significant plagioclase crystallisation, then the amphibole should have a negative Eu anomaly. This implies that the invading melt was yet to undergo plagioclase saturation, or that the melt was more water-rich and oxidising, leading to the suppression of the Eu anomaly in high An plagioclase (Philpotts 1970; Deering and Bachmann 2010).

**Fig. 12:**
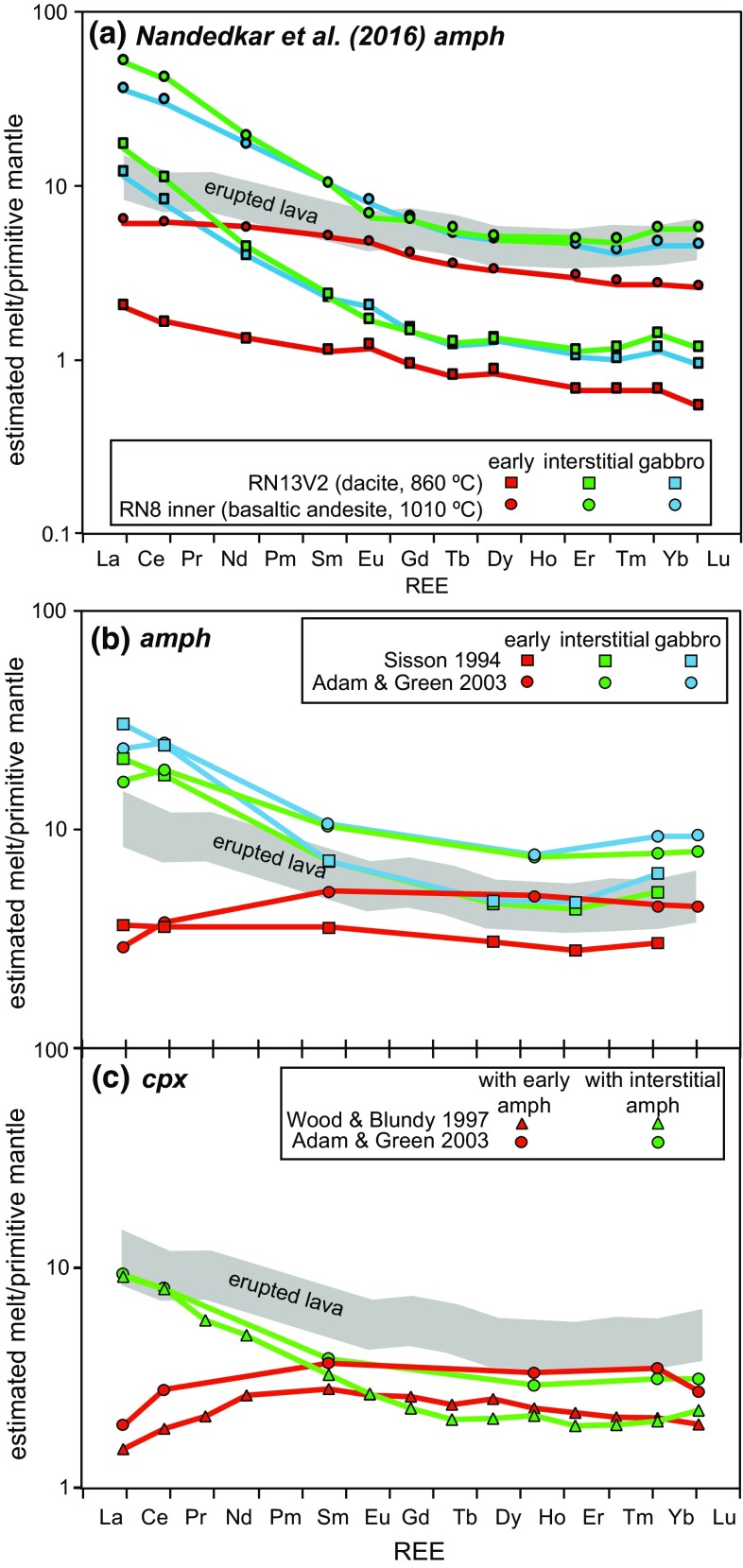
Modelled melt compositions, normalised to primitive mantle (Palme and O’Neill 2003) in equilibrium with amphibole (**a** and **b**) and clinopyroxene (**c**) types using REE partition coefficients (Sisson 1994; Wood and Blundy 1997; Adam and Green 2003; Nandedkar et al. 2016). *Grey shaded area* is the range in whole-rock REE of erupted Martinique lavas

Amphibole partition coefficients from calc-alkaline fractional crystallisation experiments of Nandedkar et al. (2016) were used to estimate melts in equilibrium with early, late and non-cumulate amphibole (Fig. 12a). Partition coefficients from a basaltic andesite (RN8 Inner), run at 1010 °C and a dacite (RN13V2) at 860 °C were chosen (Fig. 12a). Melt estimates using both experiments have the same REE profile shapes. However, higher melt REE concentrations are predicted using the partition coefficients from the basaltic andesite sample. Melts predicted using the late, interstitial amphibole has similar MREE-HREE pattern as the erupted lavas, but is significantly more enriched in LREE. In contrast, early crystallised amphibole returns melts with a flatter profile. The offset between REE concentrations predicted using the two experiments (RN8 Inner and RN13V2) highlights the extent to which amphibole partition coefficients vary with melt fractionation. Melts predicted using the partition coefficients from Sisson (1994) and Adam and Green (2003) have the same relative trends as those from Nandedkar et al. (2016) and suggest early amphibole grains crystallised from a primitive melt with a flat REE profile. The late, interstitial amphibole was crystallised from a melt enriched in LREE, similar to both the erupted lava whole-rock compositions and the melt in equilibrium with amphiboles in non-cumulate gabbros (Fig. 12b). This suggests that interstitial amphibole crystallised from evolved melts (58.8–64.6 wt% SiO_2_) which either infiltrated into a pre-exiting cumulate pile (or mush), or the erupted melts were themselves generated in, and extracted from the crystal mush.

**Fig. 13:**
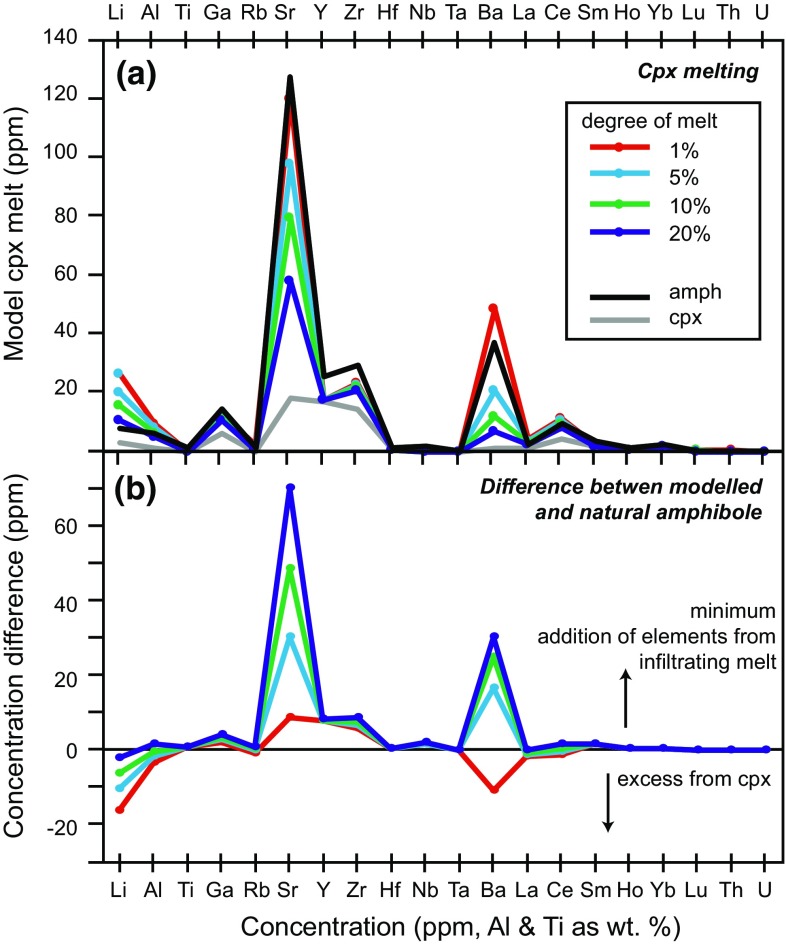
**a** Modelled trace element concentrations of the liquid produced through varying degrees of clinopyroxene melting (*coloured lines*). The compositions of clinopyroxene (*grey line*) and associated interstitial amphibole (*black line*) are shown for comparison. **b** The difference in concentration between interstitial amphibole and the liquid produced from clinopyroxene melt. Strong positive values indicate those elements that were added through an additional source such as a percolating melt

Clinopyroxene partition coefficients from Adam and Green (2003) and Wood and Blundy (1997) reveal the melts in equilibrium with clinopyroxene associated with both amphibole types have similarly contrasting REE compositions. The shape of the REE profile for melt in equilibrium with clinopyroxene associated with interstitial amphibole is very similar to the melt in equilibrium with interstitial amphibole. This suggests that a peritectic reaction occurred, in which clinopyroxene reacted with the infiltrating melt and was replaced by the interstitial amphibole. This geochemical evidence is in agreement with the textural observations (Fig. 3). However, the estimated REE concentrations are lower than both the erupted lavas and the melt in equilibrium with interstitial amphibole (Fig. 12). This observation implies that an addition of trace elements, in particular LREE into the system must have occurred after clinopyroxene crystallisation and before amphibole crystallisation. To assess the contribution of elements (Li, Al, Ti, Ga, Rb, Sr, Y, Zr, Hf, Nb, Ta, Ba, La, Ce, Sm, Ho, Yb, Lu, Th, U) from clinopyroxene to produce reaction replacement amphiboles, the melt produced from the breakdown of clinopyroxene was modelled using partition coefficients (run at 1025 °C, 0.5 GPa) from Adam and Green (2003) (Fig. 13). The chemistry of amphibole which then crystallised from the clinopyroxene melt was modelled using experimental amphibole partition coefficients (run at 1000 °C, 0.5 GPa) from Adam and Green (2003). If the modelled amphibole element concentrations were lower than natural interstitial amphibole, then an additional source of elements is needed (Fig. 13). The modal proportions of clinopyroxene and reaction replacement amphibole (e.g. MQ14; Fig. 2) suggest a significant proportion of clinopyroxene has been replaced by amphibole. Melting of up to 20 % clinopyroxene can supply the system with the concentrations of Al, Ti and the majority of trace elements needed to reproduce interstitial amphibole compositions. Apart from low degrees of meting (1 % cpx melting), a minimum additional source of Sr, Ba, Y and Zr, as well as H_2_O and Na are needed in order to produce the interstitial amphibole (Fig. 13). Therefore, a water-rich plagioclase-undersaturated melt, carrying fluid mobile elements, reacted with the cumulate/mush pile to produce late-stage interstitial amphibole. The reaction of clinopyroxene with a melt, to form amphibole has been observed in cumulates (Best 1975; Debari et al. 1987; Coogan et al. 2001; Smith 2014; Bouilhol et al. 2015) and we believe this is a common process in Martinique plutonic xenoliths.

### Open system processes

In addition to the percolating melts discussed above, plutonic xenoliths from Martinique display additional textural and chemical evidence of open system behaviour in the form of crystal cargos. One olivine gabbro sample (MQ27) shows clear evidence for the assembly of crystal cargos. Clinopyroxene cores within this sample have a larger negative Eu/Eu* of ~0.4 and a lower Mg# than the rims, which do not have a Eu anomaly and have a higher Mg# (Fig. 8). This may suggest that the clinopyroxene grains began growing from a melt that had undergone significant plagioclase crystallisation, and then interacted with and continued to grow in a lesser-evolved, plagioclase-undersaturated melt. Alternatively, the rim-forming melt may be more water-rich and oxidising, leading to the suppression of the Eu anomaly in high An plagioclase (Philpotts 1970; Deering and Bachmann 2010). Oxygen fugacity estimates from sample MQ27 amphiboles are particularly oxidising (2.2–2.9 ΔNNO; Ridolfi and Renzulli 2012) and may therefore account for the absence of a negative Eu anomaly in clinopyroxene. This evidence suggests that the plutonic xenoliths from Martinique represent the assembly of grains during transport and emplacement, and are not formed through closed system crystallisation after emplacement of a magma body. The plagioclase-undersaturated melt is consistent with the estimated composition of the percolating reactive melt described above.

The xenoliths commonly contain vesiculated interstitial glass, and few samples have a locked crystal framework. This textural evidence suggests the plutonic xenoliths are capturing the disaggregation of a crystal mush (Passmore et al. 2012). This may represent interaction at the margins of magma reservoirs stored within a larger scale mush zone. In a number of samples, edges of crystals (plagioclase and olivine) in contact with the interstitial melt have a distinctive normally zoned reaction rim of more evolved compositions. This suggests that it was an evolved melt which infiltrated a cumulate pile or mush and ‘post-cumulus’ growth of the crystals occurred prior to eruption. Within a number of samples, cross-veins of recrystallised material are present. In this case the infiltration of melt was confined to veins and did not disrupt the original crystal framework. The interstitial glass (Fig. 3a, f) contains skeletal plagioclase microlites, suggesting the host melt was rapidly quenched, and therefore the infiltration of the host melt must have occurred in a short timescale prior to eruption. Reacted rims on plagioclase are close to the compositions of plagioclase in non-cumulate gabbros (Fig. 8), and those from lavas (Pichavant et al. 2002). This provides evidence that the crystal mush interacted with the final erupted melts that are associated with non-cumulate gabbros.

Melt migration through crystal mushes is a common process within crustal storage regions (Leuthold et al. 2014; Solano et al. 2014). Exposed roots of volcanic arcs, such as the Kohistan palaeo-island arc, Pakistan (Bouilhol et al. 2015), Talkeetna, Alaska (Greene et al. 2006), Fjordland, New Zealand (Stuart et al. 2016) and exposed ultramafic complexes, Alaska (Murray 1972; Irvine 1974) allow for direct observations of the lower crust, and provide an analogue to Martinique plutonic xenoliths. In Kohistan, kilometre scale magmatic conduits are present in the lower crust, which melt rose through and reacted with the existing cumulate assemblages (Bouilhol et al. 2015). These conduits are similar to crustal feeder pipes observed in ultramafic complexes, Alaska (Murray 1972; Irvine 1974). Within these crustal sections there are outcrop scale variations in cumulate textures and crystallising phases, suggesting that it is possible to source variable plutonic xenoliths from relatively localised zones. The evidence of infiltrating melts and open system processes within Martinique cumulates suggest that they may be sourced from similar melt-rich zones, which feed shallow storage regions within the Lesser Antilles crust.

### Amphibole sponge model

Amphibole accumulation, represented by amphibole-bearing plutonic xenoliths, can be seen to have a control on the trace element signature of the lavas (Fig. 5). Amphibole preferentially incorporates MREE over HREE and therefore fractionation of amphibole with drive the melt to lower MREE/HREE (e.g. Dy/Yb) with an increasing concentration of an incompatible element (e.g. La). The fractionation of the final erupted amphibole-free lava assemblage does not have any leverage on the Dy/Yb ratio, and therefore fractionation of the cumulate assemblage can explain the variation in Dy/Yb displayed by the lavas (Fig. 6). The non-cumulate gabbro samples can be produced from the fractional crystallisation of the evolved melts generated after the initial ‘cryptic’ amphibole fractionation, and we therefore infer these samples to relate to crystallisation associated with the final erupted melt-dominant bodies stored in the shallow crust.

The data presented in this study suggest that a percolating melt through a crystal mush aided the breakdown of clinopyroxene and growth of amphibole (Best 1975; Debari et al. 1987; Smith 2014). The reactive transport of a melt through a clinopyroxene mush to crystallise amphibole will impart an amphibole fractionation signature irrespective of amphibole appearance or absence as a phenocryst phase (Smith 2014). Therefore, the presence of precursory clinopyroxene is a key requirement for the amphibole sponge in the mid-lower Lesser Antilles arc crust. The growth of interstitial amphibole as a replacement to clinopyroxene allows more interstitial liquid to be incorporated in the cumulates than would be possible in dryer basaltic systems with only amphibole-free assemblages (Meurer and Claeson 2002). Therefore, the mid-crustal storage regions provide a fertile source for melts and fluids to fuel eruptions and potentially increase the explosivity of the magmas that reach the surface.

### Plumbing system beneath Martinique

The plutonic xenoliths from Martinique used in this study provide direct evidence of open system processes, and the majority were sourced from the mid-crust and represent regions of crystal mush, within which melts are both stored and generated. Melt segregation in hot zones (Sawyer 1994; Solano et al. 2012, 2014) provides mechanisms by which a largely crystal-free melt is separated from the early formed clinopyroxene and reaction replacement amphibole-rich mushes. We can infer that the lower crust was likely a deep crustal hot zone (Annen et al. 2006). Melts were generated and stored within this region and underwent early differentiation of olivine (plus other phases) from mantle-derived primitive melts. The relatively low Fo and Ni contents of olivine provide evidence of crystallisation from a melt that has undergone prior olivine fractionation. Melts that segregate from the deep crustal hot zone ascend and stall in the mid-crust, within the plagioclase stability field. The vast majority of cumulate xenoliths from Martinique represent storage within this mid-crustal mush zone. Variations in phase assemblages may relate to localised variations in water content and temperatures within the crystal mush. Hydrous reactive melts percolate through the cumulate pile, crystallising interstitial amphibole. A large proportion of the plutonic xenoliths contain evidence for percolative melt flow and therefore likely originated in melt-rich channels within a crystal mush (Bouilhol et al. 2015). For this reason, the erupted plutonic record may be biased to sampling melt-rich zones which can eventually transport parts of the cumulate pile into the host magmas in shallow reservoirs. Crystal poor, evolved melts generated within the mid-crustal mush zone segregate and ascend to their pre-eruptive storage in shallow magma reservoirs for some time prior to eruption. The non-cumulate gabbros generally have zoned crystal phases with more evolved compositions, some amphibole-free assemblages and whole-rock chemistries which resemble erupted lavas, and therefore appear to represent the plutonic equivalents of the final erupted melts, or frozen portions of magma chambers. The ability of amphibole-rich cumulates in the mid-crust to act as a sponge provides a source for water-rich magmas, which may eventually fuel the explosive eruptions characteristic of the Lesser Antilles volcanic arc.

## Conclusions

Plutonic xenoliths from Martinique provide direct evidence for the amphibole sponge model in arc crust and the nature of the sub-volcanic plumbing system. The key findings from Martinique plutonic xenoliths are the following:

1. Crystallisation sequences of the plutonic xenoliths are variable, which could be accounted for by multiple sources of melt that differentiated at multiple depths. This observation is in contrast to plutonic xenoliths from other islands of the Lesser Antilles which have consistent crystallisation sequences.
2. All samples are inferred to be sourced from the mid-upper crust ≤15 km, at pressures of ≤4 GPa and crystallised under high water contents. Plutonic xenoliths of cumulate origin represent a mid-crustal storage region, whereas non-cumulate gabbros and gabbronorites are associated with melt-dominant bodies stored in the upper crust.
3. There is clear textural and geochemical evidence for open system processes including crystal cargos and percolating reactive melts. Therefore, plutonic xenoliths from Martinique represent mid-crustal crystal mushes in which melts can be both stored and generated.
4. Precursory clinopyroxene is a key requirement of amphibole sponge in the Lesser Antilles arc crust. Percolating melts, react with clinopyroxene to form interstitial amphibole. This is seen both texturally and in trace element concentrations of associated clinopyroxene and amphibole, where the chemistries of precursory clinopyroxene, together with an evolved percolating melt, control the composition of the later crystallised amphibole.

## Electronic supplementary material

Below is the link to the electronic supplementary material.

Excel file of Supplementary Data (XLSX 417 kb)

Zr versus Hf of amphibole and clinopyroxene from samples with either early or late crystallising amphibole, and amphiboles from non-cumulate gabbros. A large variation (nearly an order of magnitude) in incompatible trace elements is shown (PDF 90 kb)

(a) En versus Wo of orthopyroxene from different plutonic xenolith types. (b) Ti versus Ni of orthopyroxne from olivine-bearing cumulates and non-cumulate gabbros (PDF 130 kb)

Mg # versus Al_2_O_3_ and TiO_2_ of spinel from cumulates and non-cumulate gabbros (PDF 267 kb)

Coexisting plagioclase (Mol  % An) and olivine (Mol  % Fo) from plutonic xenoliths from Martinique and other islands of the Lesser Antilles. Compositions from the Lesser Antilles cover a similar range to those from other arcs worldwide (Sisson and Grove 1993b). Arrow marks a core to rim change in plagioclase An within one Martinique sample (PDF 228 kb)

Comparisons of mineral compositions in experimental troctolites, olivine gabbros, plagioclase hornblendites, hornblende gabbros, gabbronorites and hornblende gabbronorites with compositions from natural plutonic xenolith samples. Experimental run conditions from the different studies are shown. Grey shaded areas indicates the range of natural compositions in plutonic xenoliths from Grenada (Stamper et al. 2014) (PDF 686 kb)

Model estimates of temperature, oxygen fugacity and melt SiO_2_ composition for Martinique plutonic xenoliths (XLSX 46 kb)
