# Supplementary figures and images for: Plutonic xenoliths from Martinique, Lesser Antilles: evidence for open system processes and reactive melt flow in island arc crust
Source: Contrib Mineral Petrol. 2016 Sep 27;171(10):87. doi: 10.1007/s00410-016-1299-8 (PMC7175713; doi:10.1007/s00410-016-1299-8)

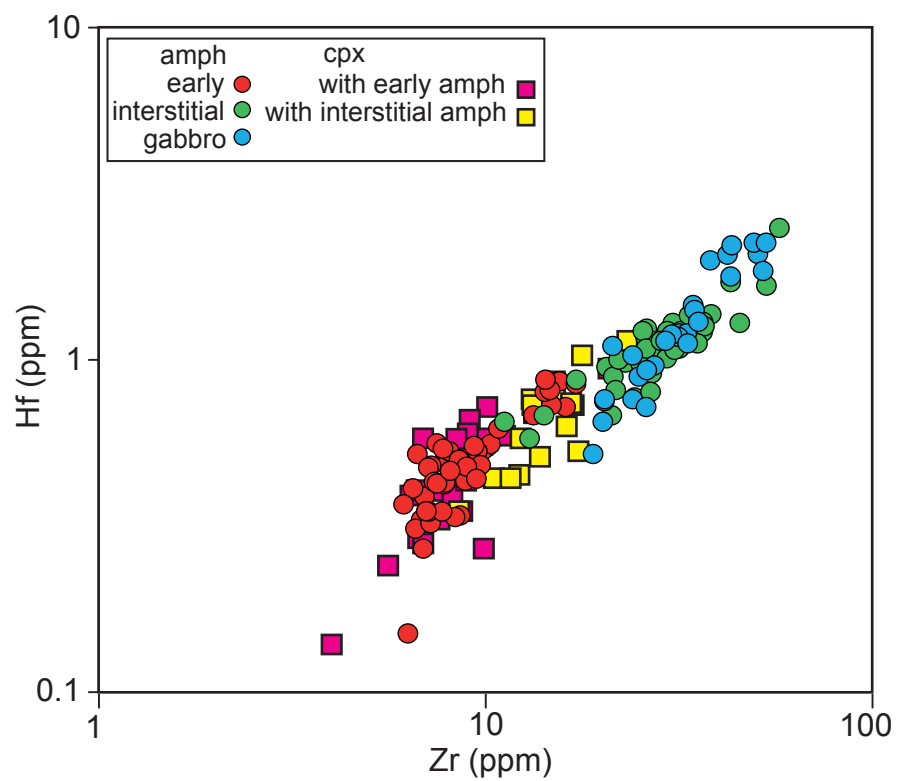

Supplement: Supplementary file 2 — Zr versus Hf of amphibole and clinopyroxene from samples with either early or late crystallising amphibole, and amphiboles from non-cumulate gabbros. A large variation (nearly an order of magnitude) in incompatible trace elements is shown (PDF 90 kb) [file 410_2016_1299_MOESM2_ESM.pdf]

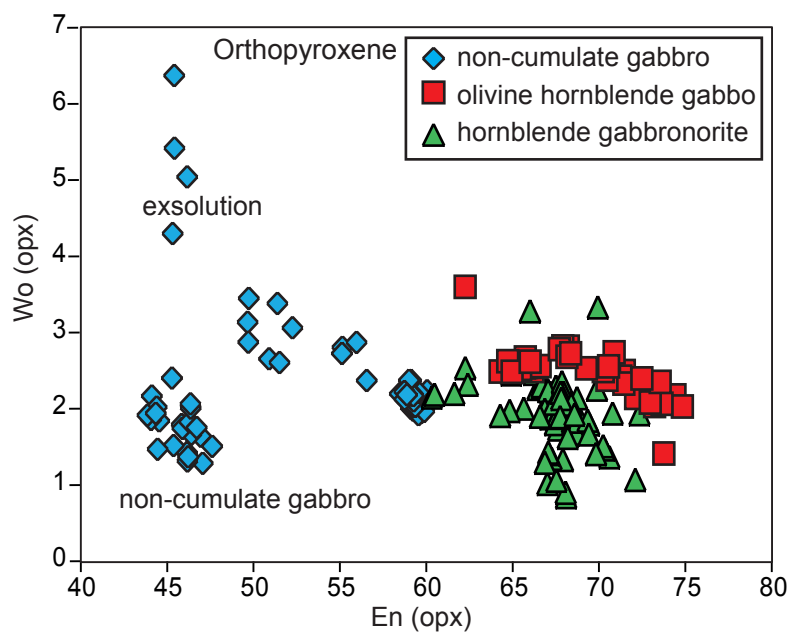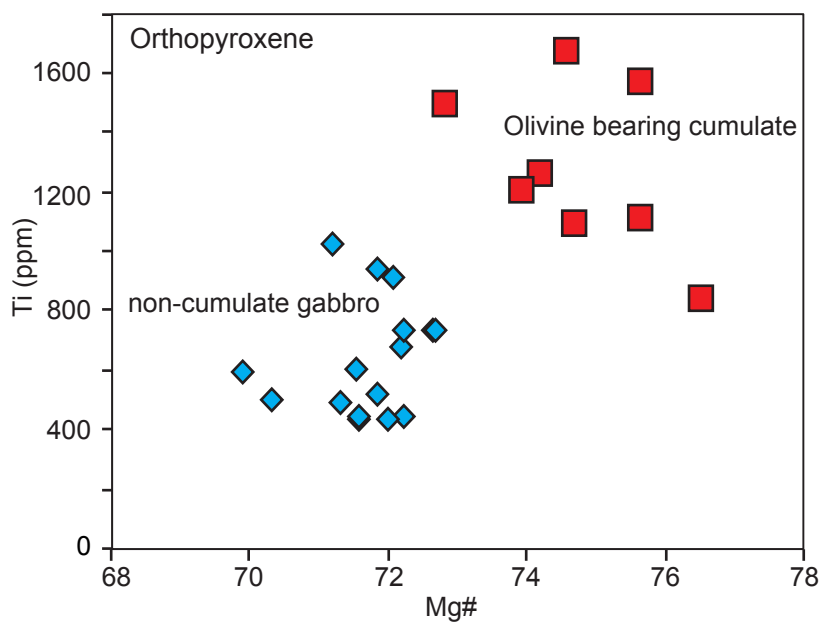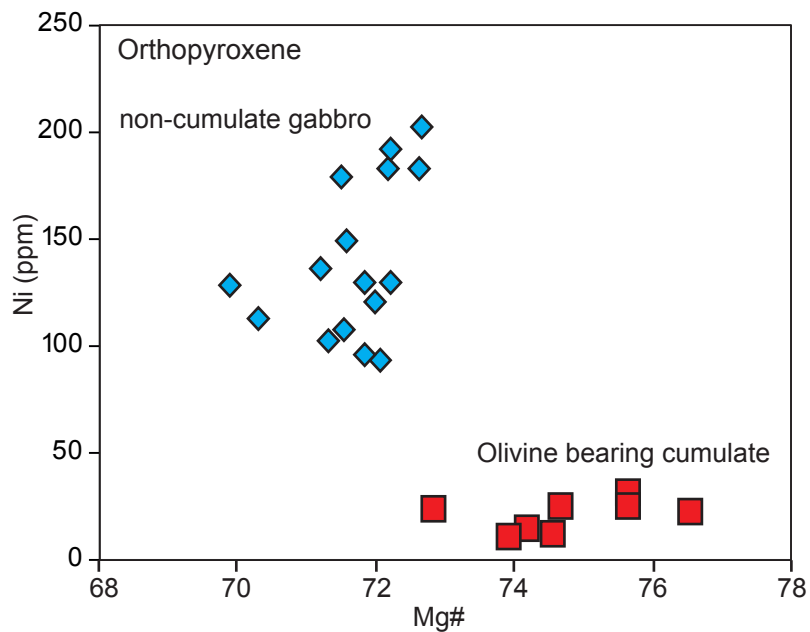

Supplement: Supplementary file 3 — (a) En versus Wo of orthopyroxene from different plutonic xenolith types. (b) Ti versus Ni of orthopyroxne from olivine-bearing cumulates and non-cumulate gabbros (PDF 130 kb) [file 410_2016_1299_MOESM3_ESM.pdf]

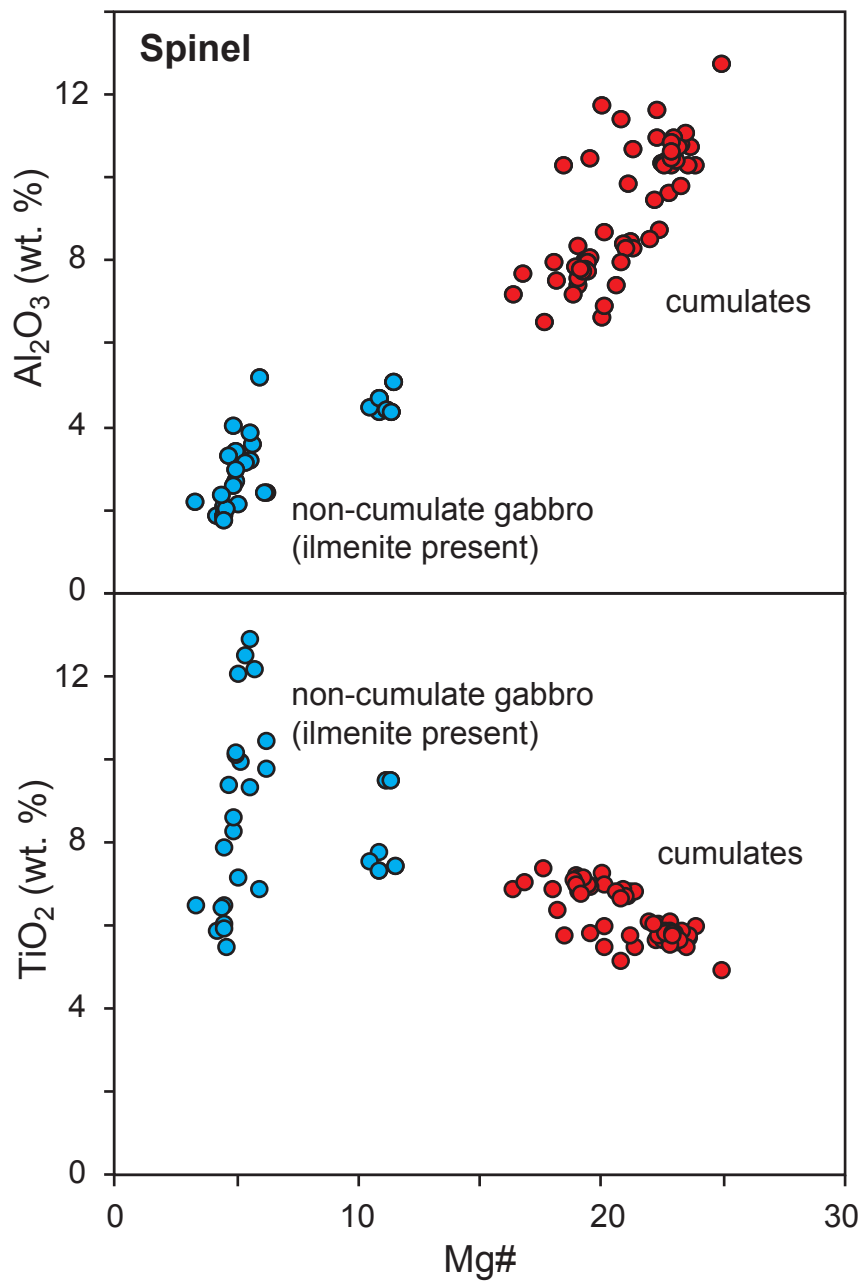

Supplement: Supplementary file 4 — Mg # versus Al2O3 and TiO2 of spinel from cumulates and non-cumulate gabbros (PDF 267 kb) [file 410_2016_1299_MOESM4_ESM.pdf]

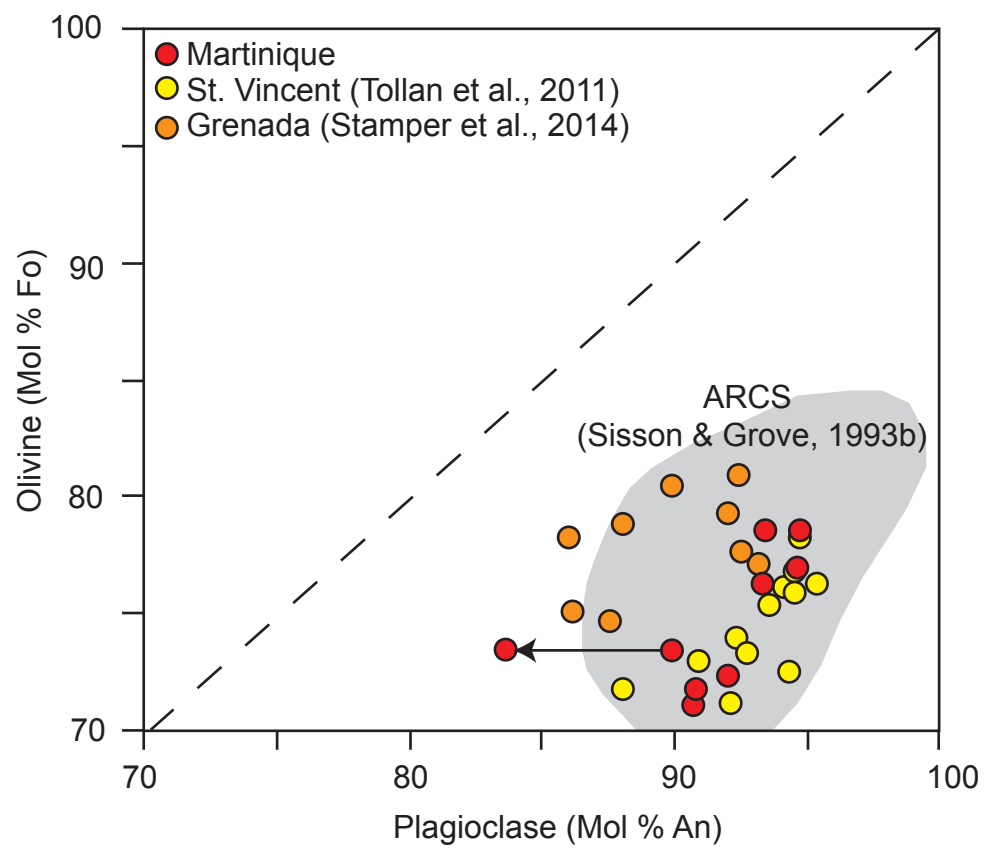

Supplement: Supplementary file 5 — Coexisting plagioclase (Mol % An) and olivine (Mol % Fo) from plutonic xenoliths from Martinique and other islands of the Lesser Antilles. Compositions from the Lesser Antilles cover a similar range to those from other arcs worldwide (Sisson and Grove 1993b). Arrow marks a core to rim change in plagioclase An within one Martinique sample (PDF 228 kb) [file 410_2016_1299_MOESM5_ESM.pdf]
