# Supplementary material for: Plutonic xenoliths from Martinique, Lesser Antilles: evidence for open system processes and reactive melt flow in island arc crust
Source: Contrib Mineral Petrol. 2016 Sep 27;171(10):87. doi: 10.1007/s00410-016-1299-8 (PMC7175713; doi:10.1007/s00410-016-1299-8)
Supplement: Supplementary file 6 — Comparisons of mineral compositions in experimental troctolites, olivine gabbros, plagioclase hornblendites, hornblende gabbros, gabbronorites and hornblende gabbronorites with compositions from natural plutonic xenolith samples. Experimental run conditions from the different studies are shown. Grey shaded areas indicates the range of natural compositions in plutonic xenoliths from Grenada (Stamper et al. 2014) (PDF 686 kb) [file 410_2016_1299_MOESM6_ESM.pdf]

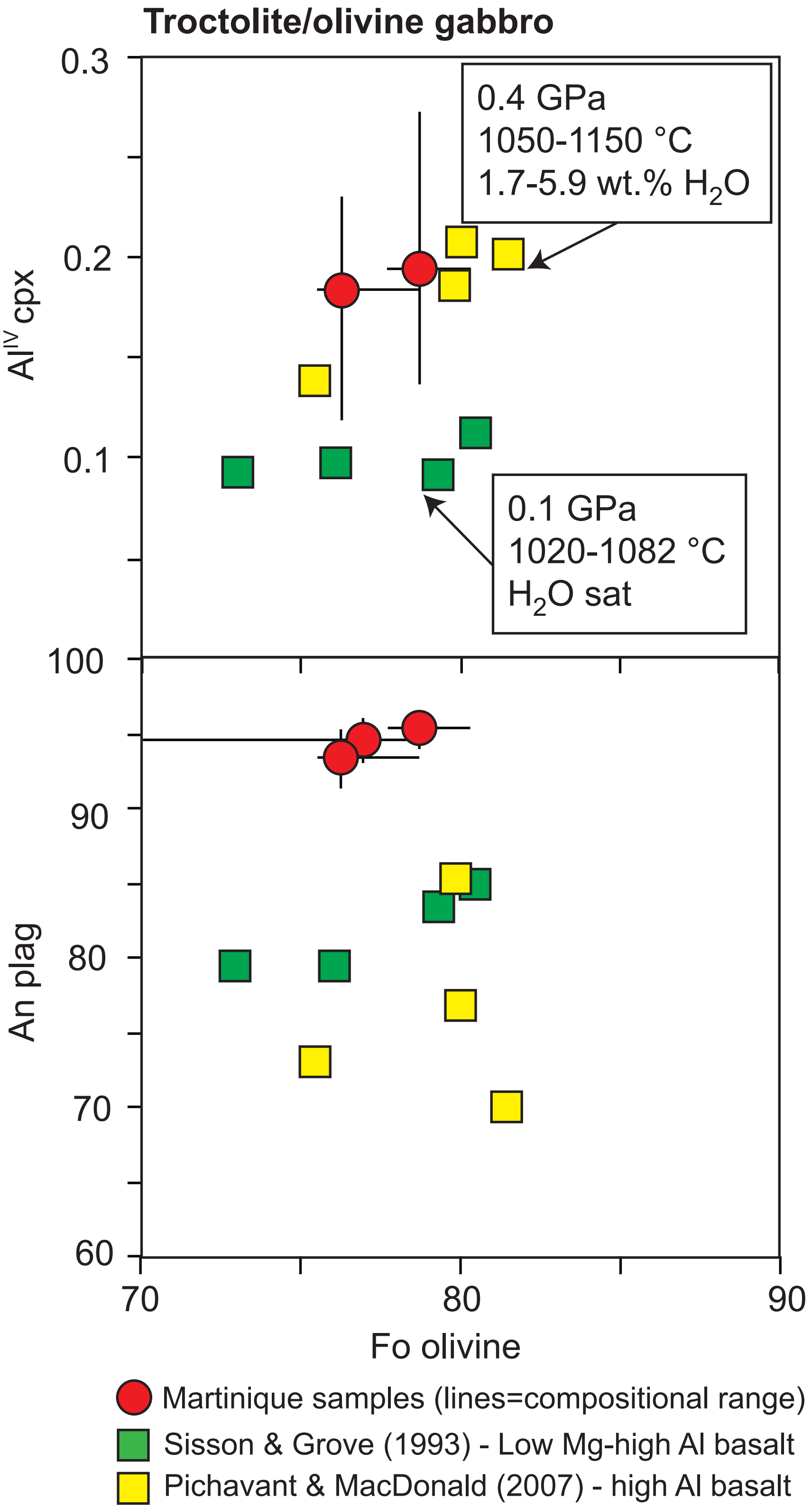
Troctolite/olivine gabbro
0.3
0.2
0.1
100
90
80
70
60
70
80
90
Al^{IV} cpx
An plag
Fo olivine
0.4 GPa
1050-1150 °C
1.7-5.9 wt.% H_2O
0.1 GPa
1020-1082 °C
H_2O sat
Martinique samples (lines=compositional range)
Sisson & Grove (1993) - Low Mg-high Al basalt
Pichavant & MacDonald (2007) - high Al basalt

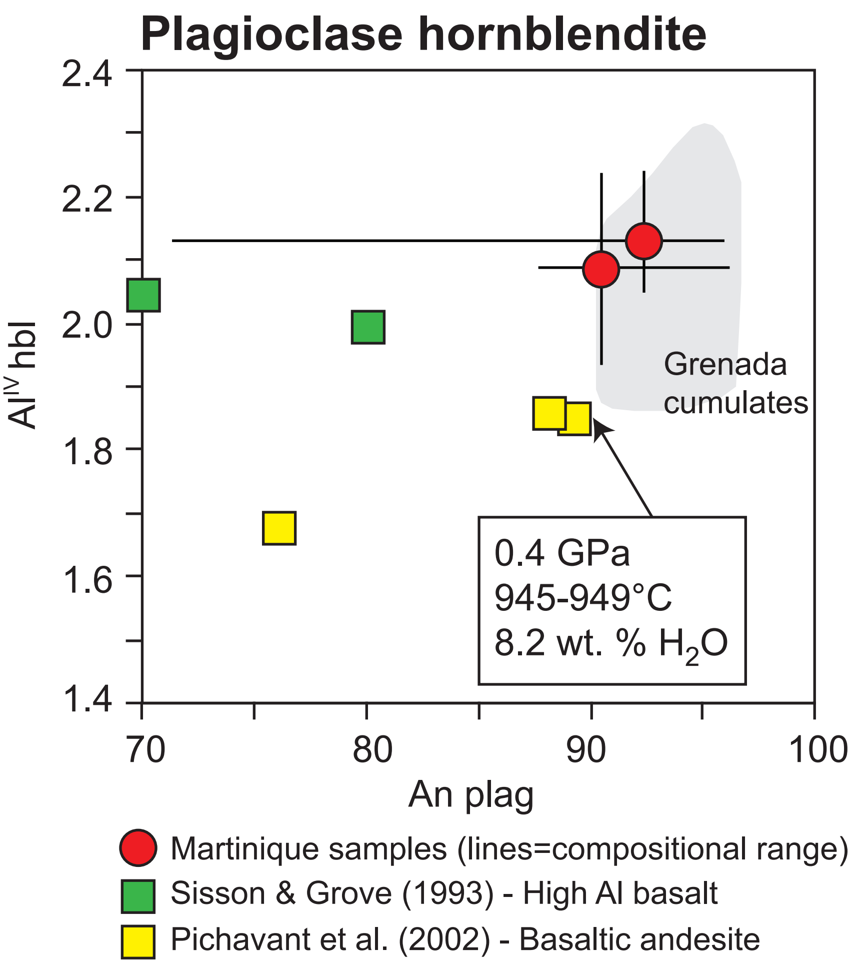

Plagioclase hornblendite
2.4
2.2
2.0
1.8
1.6
1.4
Al^{IV} hbl
70
80
90
100
An plag
Grenada
cumulates
0.4 GPa
945-949°C
8.2 wt. % H_2O
Martinique samples (lines=compositional range)
Sisson & Grove (1993) - High Al basalt
Pichavant et al. (2002) - Basaltic andesite

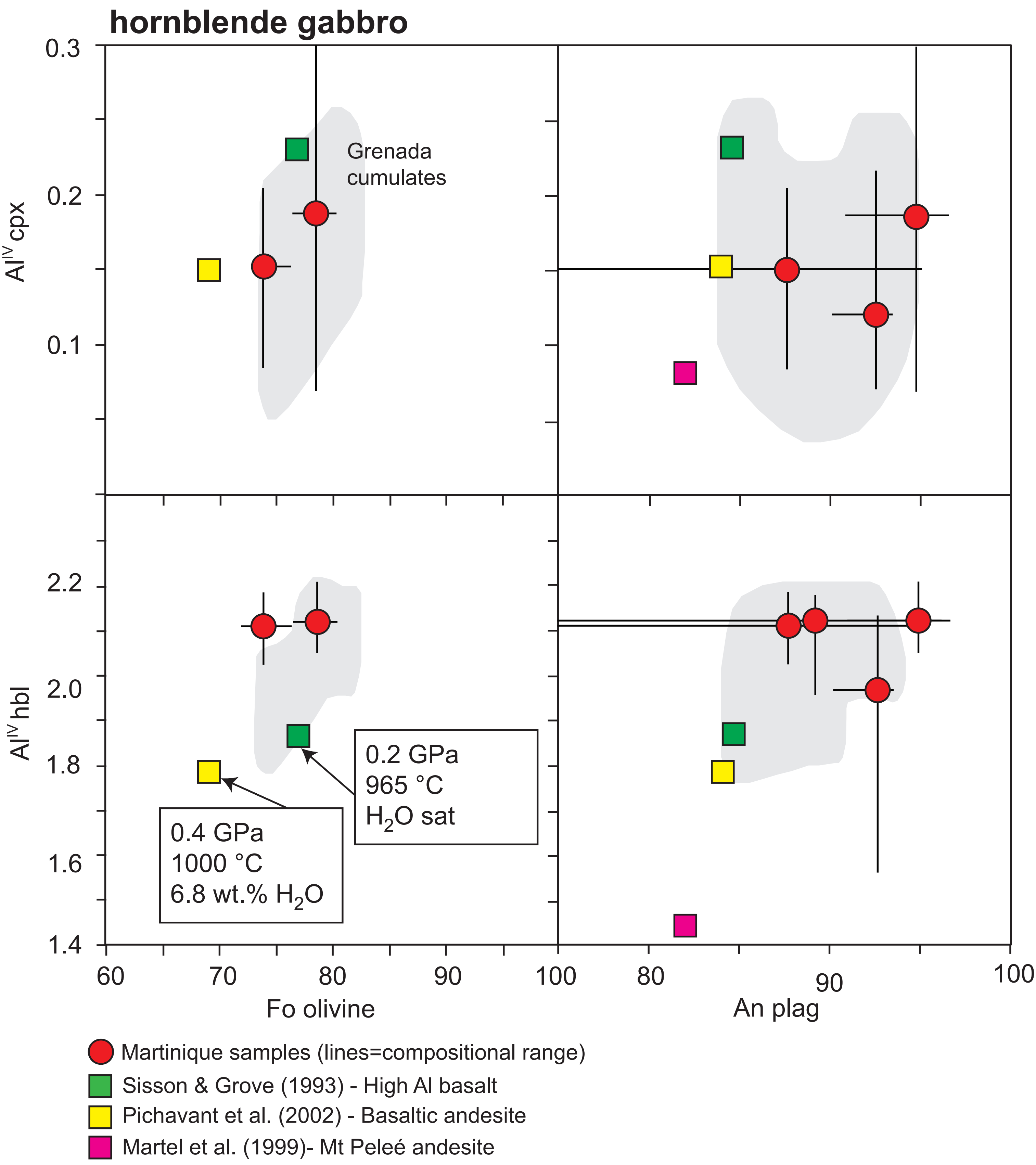
hornblende gabbro
Grenada cumulates
Al^{IV} cpx
Al^{IV} hbl
0.3
0.2
0.1
2.2
2.0
1.8
1.6
1.4
0.2 GPa
965 °C
H_2O sat
0.4 GPa
1000 °C
6.8 wt.% H_2O
60
70
80
90
100
80
90
100
Fo olivine
An plag
Martinique samples (lines=compositional range)
Sisson & Grove (1993) - High Al basalt
Pichavant et al. (2002) - Basaltic andesite
Martel et al. (1999)- Mt Peleé andesite

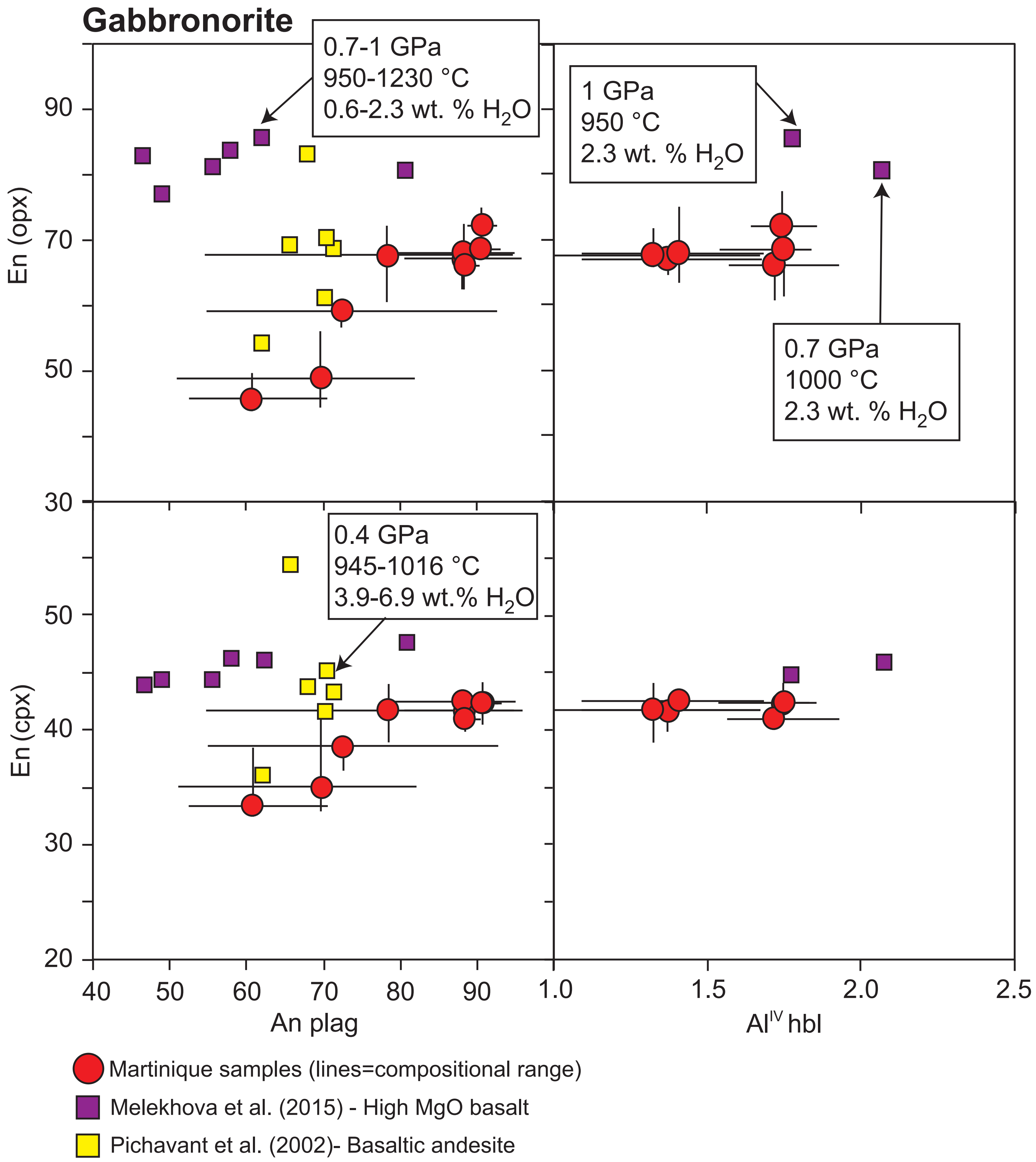
Gabbronorite
0.7-1 GPa
950-1230 °C
0.6-2.3 wt. % H2O
1 GPa
950 °C
2.3 wt. % H2O
0.7 GPa
1000 °C
2.3 wt. % H2O
0.4 GPa
945-1016 °C
3.9-6.9 wt.% H2O
En (opx)
En (cpx)
An plag
Al^IV hbl
90
70
50
30
50
40
30
20
40
50
60
70
80
90
1.0
1.5
2.0
2.5
Martinique samples (lines=compositional range)
Melekhova et al. (2015) - High MgO basalt
Pichavant et al. (2002)- Basaltic andesite
